# Polarimetric Measurements of Surface Chirality Based on Linear and Nonlinear Light Scattering

**DOI:** 10.3389/fchem.2020.611833

**Published:** 2021-02-10

**Authors:** Ankur Gogoi, Surajit Konwer, Guan-Yu Zhuo

**Affiliations:** ^1^ Department of Physics, Jagannath Barooah College, Jorhat, India; ^2^ Department of Chemistry, Dibrugarh University, Dibrugarh, India; ^3^ Institute of New Drug Development, China Medical University, Taichung, Taiwan; ^4^ Integrative Stem Cell Center, China Medical University Hospital, Taichung, Taiwan

**Keywords:** membrane, circular dichroism, optical activity, optical rotation, chirality, linear dichroism, second-harmonic generation, sum-frequency generation

## Abstract

A molecule, molecular aggregate, or protein that cannot be superimposed on its mirror image presents chirality. Most living systems are organized by chiral building blocks, such as amino acids, peptides, and carbohydrates, and any change in their molecular structure (i.e., handedness or helicity) alters the biochemical and pharmacological functions of the molecules, many of which take place at surfaces. Therefore, studying surface chirogenesis at the nanoscale is fundamentally important and derives various applications. For example, since proteins contain highly ordered secondary structures, the intrinsic chirality can be served as a signature to measure the dynamics of protein adsorption and protein conformational changes at biological surfaces. Furthermore, a better understanding of chiral recognition and separation at bio-nanointerfaces is helpful to standardize chiral drugs and monitor the synthesis of adsorbents with high precision. Thus, exploring the changes in surface chirality with polarized excitations would provide structural and biochemical information of the adsorbed molecules, which has led to the development of label-free and noninvasive measurement tools based on linear and nonlinear optical effects. In this review, the principles and selected applications of linear and nonlinear optical methods for quantifying surface chirality are introduced and compared, aiming to conceptualize new ideas to address critical issues in surface biochemistry.

## Introduction

Chirality of molecular architectures has become a subject of increasing interest to the chemical, biological, and pharmaceutical science community, largely because of its immense importance in understanding the structure and function of biological systems (Green et al., 2016; Jiang et al., 2017b; Gogoi et al., 2019; Zhao et al., 2020). Chiral compounds or molecules occur in the form of two stereoisomers that are nonsuperimposable mirror images of each other, known as enantiomers. Of particular interest is the homochirality of the left-handed amino acids and right-handed sugars which are the building blocks of the key molecules of life: proteins and nucleic acids, respectively. In fact, such homochiral selectivity is intrinsically linked to all the fundamental biochemical processes of life, including molecular recognition, synthesis, and replication, and is indeed one of the biggest enigmas of contemporary science (Toxvaerd, 2009; Blackmond, 2011; Walker, 2011; Wang et al., 2019a; Zhao et al., 2020). As far as the fullest exploitation of the unique advantages of surfaces at the nanoscale, especially in the fields of enantiomeric recognition and separation is concerned, there is a pressing need of having a molecular level understanding about how does a (chiral) molecule get adsorbed or bind itself onto a surface, membrane, or interface; whether any kind of surface modification takes place during this process and how much change of the conformation of the (chiral) molecule takes place on the surface (Gautier, 2008). Such studies are also important to shed light on the evolution of chirality across molecular to macroscopic length scales.

On the other hand, a pair of enantiomers of a chiral compound is very difficult to distinguish from each other since they possess exactly same scalar physiochemical properties, for example, density, molecular weight, melting points, boiling points, electronic and vibrational frequencies, reaction rates, and solubilities. Fortunately, their vectorial properties are an exception in the sense that these properties are sign-inverted, and the opposite enantiomers exhibit remarkably distinguishable properties when they interact with other chiral materials (e.g., other biological material, synthetic compounds, or drugs) or polarized light (Tester and Karkalas, 2003; Brunner, 2004; Tang and Cohen, 2010; Lewis, 2013). For example, enantiomers of a chiral molecule have the ability to rotate a plane polarized light by equal amounts, but in opposite directions, leading to optical rotation spectra which are mirror images of each other (Tranter, 2012; Qiu et al., 2016). Depending on the rotation produced in an ideal environment, a chiral molecule can be designated as dextrorotatory (if the rotation is positive or right-handed or clockwise) or levorotatory (if the rotation is negative or left-handed or counterclockwise) enantiomer. This property of a chiral molecule is also known as circular birefringence (CB) since it occurs due to the different indices of refraction for right circularly polarized (RCP) light and left circularly polarized (LCP) light (Liehr, 1964). Likewise, a chiral substance also possesses different absorption cross sections for RCP and LCP, an effect known as circular dichroism (CD) (Fischer and Hache, 2005; Tang and Cohen, 2010). Manifestation of molecular chirality can also be observed in other forms of optical activities, such as Raman optical activity (ROA) and circularly polarized luminescence (CPL).

Admittedly, molecular chiroptical signals are usually weak due to the large difference between the molecular scale and the probing wavelength. Therefore, in addition to molecular chirality, optical activities at surfaces (equivalent to interfaces in this review) are gaining much attention due to the possibility to acquire dramatically enhanced chiroptical interactions and probe chirality and/or changes in chirality at such surfaces (Byers et al., 1994a; Verbiest et al., 1995; Hicks and Petralli-Mallow, 1999; Kneipp et al., 2010; Ben-Moshe et al., 2013). Such studies are also important for their potential to elucidate the underlying process of chiral recognition at surfaces (Ernst, 2006; Gogoi et al., 2019). Although the linear optical activities, as mentioned above, have been used to study surface chirality, they are often limited by surface specificity (or monolayer sensitivity) and concentration, resulting in weak response, that is, surface insensitive. On purpose of compensating the weaknesses of linear optical methods, nonlinear optical methods, including second-harmonic generation (SHG) and sum-frequency generation (SFG), are proposed due to the characteristics of label-free, noninvasive detection and surface specificity (Shen, 1984; Byers et al., 1994a; Shen, 1994; Shen, 1996; Vogel, 1996; Fischer and Buckingham, 1998; Verbiest et al., 1998). SHG and SFG are contrast mechanisms exclusively sensitive and specific to noncentrosymmetric structures, for example, surface, molecular chirality, and chiral molecules adsorbed on a surface. Thus, using SHG or SFG to study surface chirality is a background-free probe because an achiral liquid cannot contribute to the signal, which is named as C-SHG or C-SFG hereafter. Unlike the OA effects measured by linear optical methods that are mainly contributed from magnetic dipole or quadrupole interactions, C-SHG and C-SFG are electric dipole–allowed transitions (Petralli-Mallow et al., 1993; Byers et al., 1994a; Ji and Shen, 2006), manifesting that they are correlated with a unique molecular structure and a polarized excitation (Simpson, 2002). For this reason, the measurement techniques are designed with specific polarization settings and experimental geometry. Combined with the structural requirements for C-SHG and C-SFG, related techniques are well applicable to selectively probe surface chirality.

Since there are few articles on systematic discussion and comparisons for the abovementioned methods used for the interrogation of surface chirality, in this review, a clear and comprehensive view of polarimetric measurements of surface chirality is presented using several classic articles. The content is started from the linear optical activities, for example, CB, CD, and ROA, and then provides a closer look at the nonlinear optical methods, for example, C-SHG and C-SFG, and their applications. We will deliberately concentrate on the applications of linear and nonlinear chiroptical methods, instead of the development of theory, in studying the chirality at liquid, solid, film, and nanoparticle (NP) surfaces, air/liquid, liquid/liquid, and solid/liquid interfaces, membranes, etc. in the following sections.

### Linear Optics on Surface Chirality

Optical techniques offer unique opportunities for the study of multiscale chirality ranging from molecules to surfaces. Notably, the discovery of chiroptical activities dates back to 1848 when Pasteur observed chiral asymmetry in the form of optical rotation in the isomers of tartaric acid (Flack, 2009; Gal, 2013). Over the past century, the constant evolution of chiroptical techniques, such as, optical rotatory dispersion (ORD), electronic circular dichroism (ECD), vibrational circular dichroism (VCD), and Raman optical activity (ROA), has provided unprecedented insight into the chiral signature of molecules, monolayers, or surfaces, especially in the fields of pharmaceutical, agrochemical, and flavor and fragrance technology (Collins et al., 2017; Ozcelik et al., 2020). In addition, there are few other linear optical techniques, such as Rayleigh optical activity (Andrews, 1980; Hoffman et al., 1994; Cameron and Barnett, 2014), circular differential Mie scattering (Yoo and Park, 2015), and Mueller matrix ellipsometry (Podraza et al., 2008), which are used for the interrogation of chiral structures. As yet, despite showing promising results, these techniques have not found widespread applications for the study of surface chirality. This section will cover the fundamentals and highlight the recent applications of the linear chiroptical techniques that are widely used for interrogating chiral surfaces.

### Optical Rotation and Optical Rotatory Dispersion

The optical rotation by a chiral substance is probably the most commonly known chiroptical activity, which is actually the consequence of circular birefringence (CB). Briefly, when a beam of plane polarized light (which may be considered as the resultant of two opposite circularly polarized components of same wavelength and intensity) enters a (optically active) chiral medium, the RCP and LCP experiences different indices refraction resulting in a phase shift between the orthogonal polarizations. Consequently, both the RCP and LCP components will travel at different velocities through the chiral medium, thereby continuously rotating the plane of their resultant electric field vector, a phenomenon similar to linear birefringence (Liehr, 1964; Snatzke, 1968; Kumar and Liz-Marzán, 2019). Now since refractive index is a function of wavelength of the incident light (optical dispersion), optical rotation is also dispersive in nature. Importantly, when measured as a function of incident wavelength, OR is termed as optical rotatory dispersion (ORD). Notably, OR and ORD are represented by specific rotation, *α*, and molar rotation, *M*, respectively (Edwards and Jenkinson, 2012).

OR and ORD has been extensively used for stereochemical assignment, determination of enantiomeric purity of products emerging from asymmetric synthesis, glucose concentration, studying molecular structures (particularly secondary structures of large molecules like proteins), etc (Djerassi et al., 1956; Cantor et al., 1966; Magar, 1968; Snatzke, 1968; Chen et al., 1972; Tranter, 2012; Stark et al., 2019). From the instrumentation point of view, several efficient methods for the measurement of optical rotatory dispersion spectrum have been reported (Castiglioni et al., 2011; Han et al., 2014; Jiang et al., 2017a; Goldberg and Weissman, 2019; Liu et al., 2020). Most recently, a novel technique by combining surface plasmon resonance (SPR) and weak value amplification (WVA) was used for high resolution measurements of extremely small optical rotation of chiral molecules in dilute solutions (Xu et al., 2020). In addition to static ORD, several time-resolved ORD experiments have also been demonstrated (Shapiro et al., 1995; Chen et al., 2003; Chen et al., 2007). A number of models have also been developed in the recent past to theoretically predict OR and ORD (Polavarapu, 2002).

**FIGURE 1:**
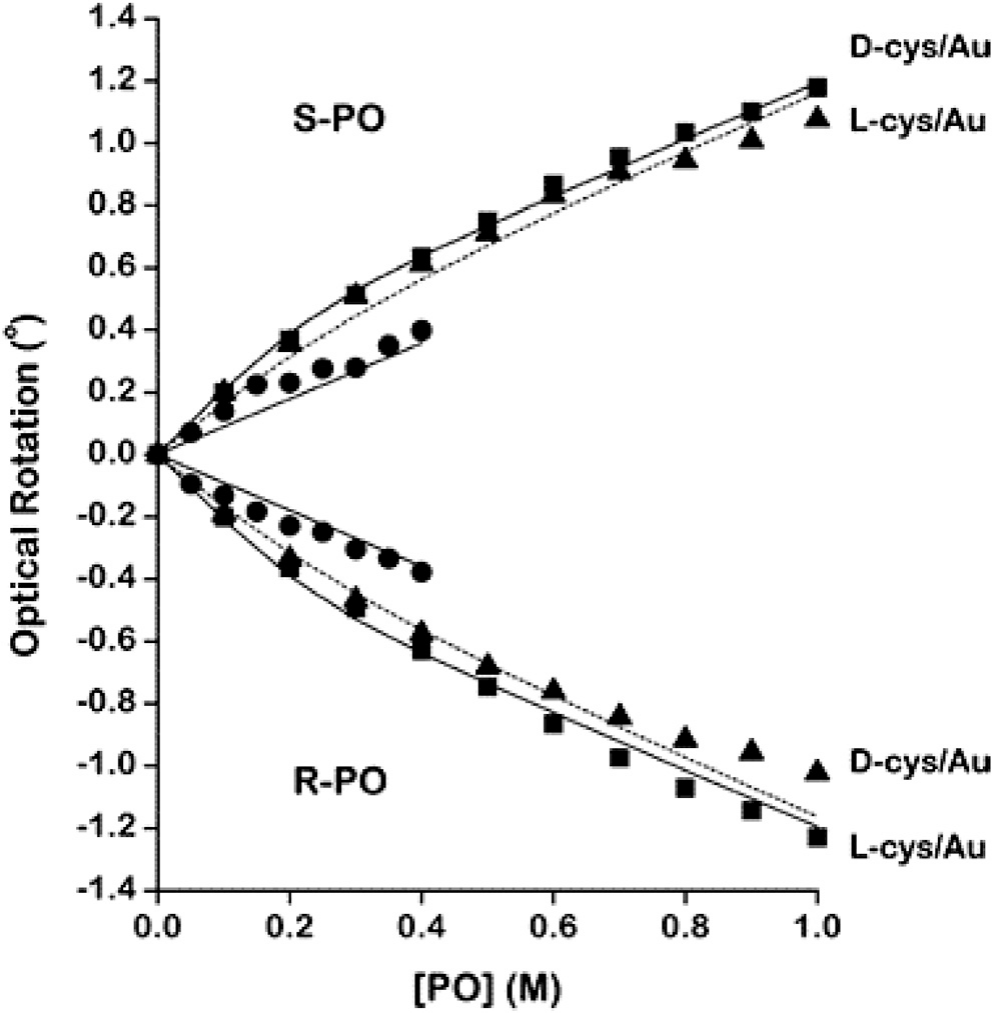
Optical rotation by (*R*)- and (*S*)-PO in pure aqueous solutions (●) and in solutions containing Au nanoparticles modified with D- and L-cysteine. The data from the solutions containing the heterochiral pairs ((*R*)-PO/L-cys and (*S*)-PO/D-cys) are marked with solid squares (■). The data from solutions containing the homochiral pairs ((*R*)-PO/D-cys and (*S*)-PO/L-cys) are marked with solid triangles (▲). The solid and dashed lines mark the fits of the adsorption model described in the text. The deviations between the data for the homochiral and the heterochiral pairs are indicative of the enantiospecific interaction of (*R*)- and (*S*)-PO with the chiral Au nanoparticles. The figure and caption have been adapted with permission from Shukla et al., (2010), Copyright ^©^ 2010 American Chemical Society.

Apart from its application in studying molecular chirality, OR and ORD have also been used to study the chirality of nanostructures, membranes, and surfaces. For example, Lenard and Singer, (1966) conducted room temperature ORD measurements to elucidate the structure of membranes made of human red blood cells and of *B. subtilis*. The obtained spectra revealed that a part of the membrane protein is in a helical conformation, while the remainder was primarily in a random coil form. Green et al., (1973) studied the transfer or exchange of cholesterol from phospholipid molecules in liposomes to membranes of human erythrocyte by measuring ORD curves. In a recent work, Esposito et al., (2015) experimentally compared the ORD of different helical nanostructures, such as single helical nanowires (SHN), closely packed single helical nanowires (CP-SHN), and triple helical nanowires (THN). The group observed ORD up to 8° for THN which was independent of the sample orientation with respect to the incoming linear polarization in a broad spectrum ranging from 800 nm to 1050 nm. Conversely, orientation and wavelength-dependent optical activities were observed in cases of SHN. Such tailored chiroptical properties are particularly relevant in the fields of enantio-detection in medical and biological sciences as well as nanophotonics, spintronics, etc. In another work, the ability of polarized near-field scanning optical microscopy (NSOM) in determining the near-field OA of chiral crystal surfaces of enantiomerically pure amino acids was demonstrated (Dressler et al., 2007). Furthermore, Shukla et al. (Shukla et al., 2010; Shukla et al., 2014) used optical rotation measurements to demonstrate the enantioselective absorption of chiral probes such as *R*- or *S*-propylene oxide (PO) by Au NPs coated with D- or L-cysteine (cys). Impressively, the group fabricated chiral Au NPs by modifying the surface with racemic cys or enantiomerically pure D- or L-cys. Subsequently, polarimetry was used to probe the adsorption of racemic PO and enantiopure *R*- or *S*-PO onto these modified Au NPs (Shukla et al., 2010). Figure 1 shows the OR measurements during the interaction of surface modified Au NPs with *R*- or *S*-PO. Moreover, the group studied the influence of wavelength, temperature, and NP size on the magnitudes of optical rotation measurements obtained from the enantiospecific interaction of D- or L-cys–modified Au NPs during addition of racemic (both *R*-PO and *S*-PO) PO (Shukla et al., 2015). Advancing their prior works, the group further exploited the capability of OR measurements to demonstrate the enantioselective binding of *R*- or *S*-propranolol, one of the well-known pharmaceuticals, onto the chirally modified surfaces of tetrahexahedral (THH, 24-sided) Au NPs (Shukla et al., 2016). Chirality of chitosan (a type of biocompatible and biodegradable chiral aminopolysaccharide), on the other hand, has attracted much attention in the recent years due to their potential application in medicine, pharmacology, and tissue engineering (Croisier and Jérôme, 2013; Gegel et al., 2018a; Gegel et al., 2018b). For instance, Shipovskaya et al., (2017) reported the optical activity possessed by films of chitosan having a broad range of molecular masses by conducting ORD measurements. The group observed the modulus of optical activity [*α*] of the films is controlled mainly by chemical structure and molecular arrangement in the film. In addition, significant variation in the measured values of [*α*] of thermally modified chitosan films was also observed in the study. Following this, the group further reported the optical activity anisotropy of chitosan films (Shipovskaya et al., 2019).

### (Electronic) Circular Dichroism

Circular dichroism (CD) is the manifestation of the differential absorption of RCP and LCP by a chiral media. It can occur in all wavelength ranges resulting in electronic circular dichroism (ECD) or vibrational circular dichroism (VCD), depending whether the probing wavelength is in the ultraviolet (UV), visible, or infrared (IR) spectral range, respectively (Zsila, 2010; Tranter, 2012). Notably, OR/ORD and CD are complementary to each other and are interwoven through Kramers–Kronig relations (Emeis et al., 1967; Krykunov et al., 2006; Kumar and Liz-Marzán, 2019). Nevertheless, there is a basic difference between OR/ORD and CD from classical electromagnetic point of view. OR/ORD arises due to the difference in the real part of the complex refractive index which affects the phase velocities of the RCP and LCP of the incident electromagnetic radiation, while the origin of CD can be attributed to the differences in the imaginary part of the refractive index which affects the extinction coefficients (Narushima and Okamoto, 2016; Collins et al., 2017; Kumar and Liz-Marzán, 2019; Yoo and Park, 2019). Importantly, for a medium exhibiting both ORD and CD, the RCP and LCP components of an originally linearly polarized radiation will suffer unequal absorption (i.e., the magnitude of the electric field vectors of the RCP and LCP components leaving the medium will be different), leading to an elliptically polarized resultant beam emerging out of the medium, the major axis of which will define the rotated direction of the new plan of polarization (Liehr, 1964; Snatzke, 1968; Yoo and Park, 2019). Typically, the elliptically polarized beam after suffering CD is characterized by the ellipticity parameter, *θ* measured in degrees (Tranter, 2012).

CD is indeed one of the most straightforward yet powerful chiroptical methods due to its unique advantages, such as simple operating procedure, requirement of small concentration (10^−5^ M) of samples, and rapid data acquisition (few milliseconds for single-wavelength measurements) enabling to monitor dynamic events (Zsila, 2010; Rodger, 2013; Petrovic et al., 2015). CD has been used extensively for studying the configurational and conformational features of bioorganic molecular structures (Greenfield, 1999; Berova et al., 2007; Ranjbar and Gill, 2009; Gopal et al., 2012).

**FIGURE 2:**
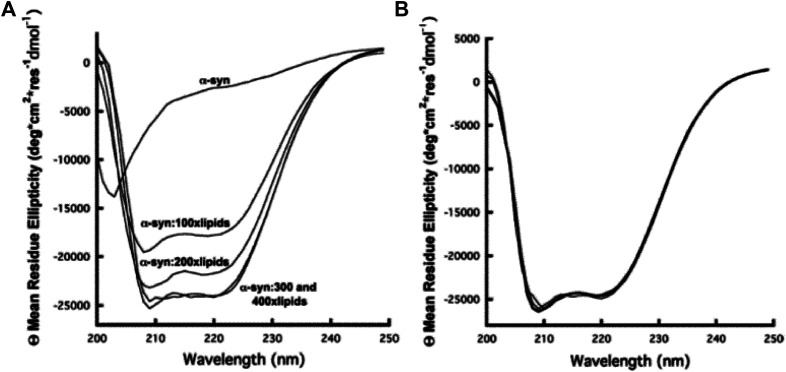
**(A)** CD spectra of *α*-syn as a function of addition of lipid in the form of SUVs. From top to bottom, 10 μM *α*-syn without lipids, 10 μM *α*-syn with 1 mM lipids, 10 μM *α*-syn with 2 mM lipids, 10 μM *α*-syn with 3 mM lipids, and 10 μM *α*-syn with 4 mM lipids, respectively. The CD signal intensity reaches a maximum at a lipid: *α*-syn molar ratio of 300:1. **(B)** Cu^2+^ titration of the 10 μM *α*-syn−3 mM lipid complex. Cu^2+^ concentrations of 0, 10, and 100 μM give overlapping *α*-syn CD spectra. The figure and caption have been adapted with permission from Dudzik et al., (2013), Copyright ^©^ 2012 American Chemical Society.

Notably, a number of studies aimed at revealing the architecture of biological membranes have been reported (Schneider et al., 1970; Urry et al., 1971; Litman, 1972; Urry and Long, 1974; Long et al., 1977). However, in many cases, application of CD to study the structure and function of biomembranes becomes cumbersome because of the distorted spectra originated due to the scattering by the membrane particulates (Urry and Long, 1974; Gitter-Amir et al., 1976; Long et al., 1977). On the other hand, significant number of efforts have been devoted for the study of structure and function of proteins by using CD, mainly because of its immense importance in the fields of proteomics and structural genomics (Kelly and Price, 2000; Greenfield, 2004; Kelly et al., 2005; Greenfield, 2006; Micsonai et al., 2015). In addition, several online tools and databases have also been developed for the analysis and deposit of CD spectra of protein structures (Whitmore and Wallace, 2004; Whitmore and Wallace, 2008; Abdul-Gader et al., 2011; Whitmore et al., 2011; Micsonai et al., 2015). Of paramount importance are the membrane proteins, which represent approximately 30% of all proteins of human genome sequenced so far. Such proteins take part in vital physiological functions including molecular recognition, cell adhesion, etc., and, most importantly, single-pass membrane proteins alone constitute around 69% of current approved drug targets (Sood and Gross, 2019). Notably, such proteins are not only difficult to crystallize for X-ray crystallographic studies but also a difficult target for nuclear magnetic resonance (NMR) spectroscopy. In this regard, CD spectroscopy has become one of the most reliable choices to uncover valuable information on the structure, function, and dynamics of membrane proteins (Sreerama and Woody, 2004; Siligardi et al., 2014; Matsuo et al., 2016; Miles and Wallace, 2016). In a time-resolved CD spectroscopy measurement, Horn et al. (Horn et al., 2007) successfully studied the kinetic phases of light-harvesting chlorophyll a/b (LHCIIb) protein complex. Dudzik et al., (2013) used far-UV CD spectroscopy to investigate the coordination of copper to the membrane-bound form of *α*-synuclein (*α*-syn), a neurological copper-binding protein related to Parkinson’s disease. The study revealed that there was no change in the membrane-bound *α*-syn helicity after binding with a single equivalent of Cu^2+^ at its N-terminus, as shown in Figure 2. In another study, combination of vacuum ultraviolet circular dichroism (VUVCD) and linear dichroism (LD) spectroscopy was used to study the conformation as well as the interaction mechanism of phosphatidylglycerol liposome membrane–bound a-lactalbumin (LA), thioredoxin (Trx), and b-lactoglobulin (LG) proteins (Matsuo et al., 2016). Notably, the structure and function of such membrane anchored or adsorbed proteins are inextricably related to each other, and their proper study may lead to the better understanding of the evolution of proteins to have novel and/or specific functions (Thyparambil et al., 2015).

Moreover, a number of CD techniques were reported for interrogating the change of peptide and protein conformation on interacting with different surfaces/monolayers, which can be achieved by comparing the CD spectra before and after interaction (Mcmillin and Walton, 1974; Vermeer and Norde, 2000; Burkett and Read, 2001; Fears et al., 2009; Sivaraman et al., 2009). For example, McMillin et al. (Mcmillin and Walton, 1974) demonstrated a CD technique to study the structural change of two blood proteins bovine fibrinogen and clotting factor XII (Hageman factor) adsorbed on a quartz surface. Tanaka et al., (2000) studied blood compatibility of different polymer surfaces, for example, poly(2-methoxyethylacrylate) (PMEA) and poly(2-hydroxyethylmethacrylate) (PHEMA), by using CD to examine the conformational change of surface-adsorbed bovine serum albumin (BSA) and human plasma fibrinogen (FNG). In another work, CD spectra of ultrathin films of the immobilized proteins, RC-b562 and NC-b562, on bare and modified gold surfaces were utilized to study the protein secondary structures. The group found that the secondary structures of the covalently anchored RC-b562 on modified gold substrates were preserved as compared to that of adsorbed NC-b562 molecules, which were found to be aggregated (Shimizu et al., 2003). Moreover, conformation of melectin and antapin antimicrobial peptides was studied in environments simulating eukaryotic and bacterial membranes by using CD spectroscopy (Kocourková et al., 2017). Furusawa and Kan, (2020), on the other hand, evaluated the ECD spectra of a single-layered circular dichroic film constructed by transferring Au nanospiral structures onto a flexible and transparent polydimethylsiloxane (PDMS) thin film. Excitingly, the results suggested the applicability of the film as a circular dichroic filter having CD peak of −830 mdeg at 630 nm.

Regarding chiral nanostructures, metallic NPs, metal oxide NPs, carbon nanostructures, semiconductor NPs, etc. possess excellent biocompatibility and thus have tremendous potential in biomedical applications (Zhao et al., 2020). Such nanostructures exhibit unique chiroptical properties, including CD which is mainly used to study the conformation and denaturation of surface-adsorbed molecules (Liu and Webster, 2007; Ranjbar and Gill, 2009; Ma et al., 2017; Puri and Ferry, 2017; Lu et al., 2018; Chen et al., 2019; Kuznetsova et al., 2019; Mokashi-Punekar et al., 2019; Zhu et al., 2020). For instance, conformational change of DNA structure due to the interaction with quaternary ammonium-functionalized (Goodman et al., 2006) and amine-terminated cationic (Ghosh et al., 2007) NPs was investigated by CD. Importantly, such studies are of paramount importance in the field of clinical medicine for nucleic acid delivery (Chowdhury and Akaike, 2005). Recently, Slocik et al., (2020), while studying the interaction of chiral peptides with Au NPs by using CD, demonstrated that Au NPs are capable of restructuring the chirality of D-peptides. Most recently, chiroptical properties (CD and CPL) of CdSe/CdS nanocrystals of different anisotropic shapes were investigated to understand the effect of morphology on the surface ligand–induced chirality (Hao et al., 2020).

**FIGURE 3:**
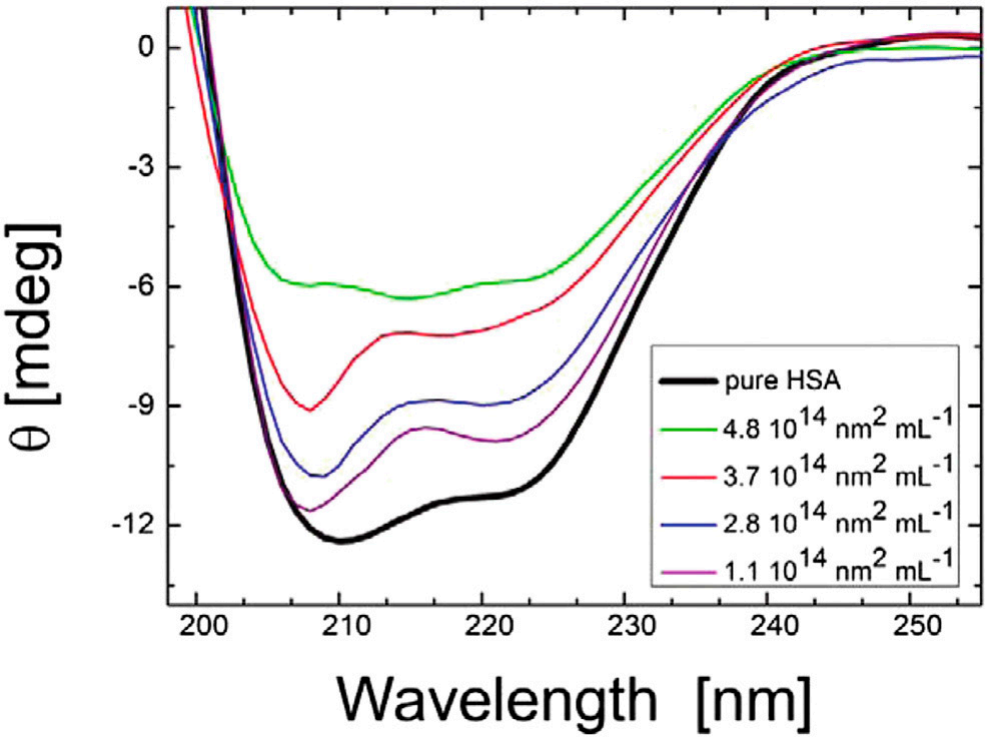
CD spectra of HAS with citrate-stabilized silver nanoparticles (diameter of 36 nm with NP surface site concentrations ranging from 1.1 × 1014 to 4.8 × 1014 nm^2^ mL^−1^ and a protein concentration of 0.052 mg mL^−1^). The figure and caption have been adapted with permission from (Gebauer et al., 2012), Copyright ^©^ 2012 American Chemical Society.

In addition, NPs strongly interact with biological fluids such as blood, plasma, or intracellular fluids, leading to the creation of protein corona due to the adsorption of proteins on the NP surface (Mahmoudi et al., 2011; Liu et al., 2013; Salvati et al., 2013; Pederzoli et al., 2017). Importantly, the formation of protein corona not only changes the surface properties of NPs but also affects the structure and function of the adsorbed proteins (Wang et al., 2017). In this context, CD has been used extensively to study the changes in the structures of the proteins and their folding and binding properties after forming corona on NP surfaces (Venerando et al., 2013; Fleischer and Payne, 2014; Zhao et al., 2015; Jayaram et al., 2018; ZhangT. et al., 2019; Li and Lee, 2020; Marichal et al., 2020; Park, 2020). Impressively, Gebauer et al., (2012) investigated the protein corona formed by human serum albumin, containing *α*-helices as predominant secondary structural elements, around citrate-functionalized silver NP surface. As shown in Figure 3, CD spectroscopy successfully revealed the structural changes in the adsorbed protein. In another work, Wang et al., (2017) used CD spectroscopy to study the transferrin (Tf) protein corona formation onto the surface of gold NPs (Au NPs), functionalized with D, L, and racemic isomers of penicillamine. The change in the secondary structures of Tf due to the formation of corona on the surface of Au NPs was confirmed by the observed variation in the values of ellipticity and other features of CD spectra.

**FIGURE 4:**
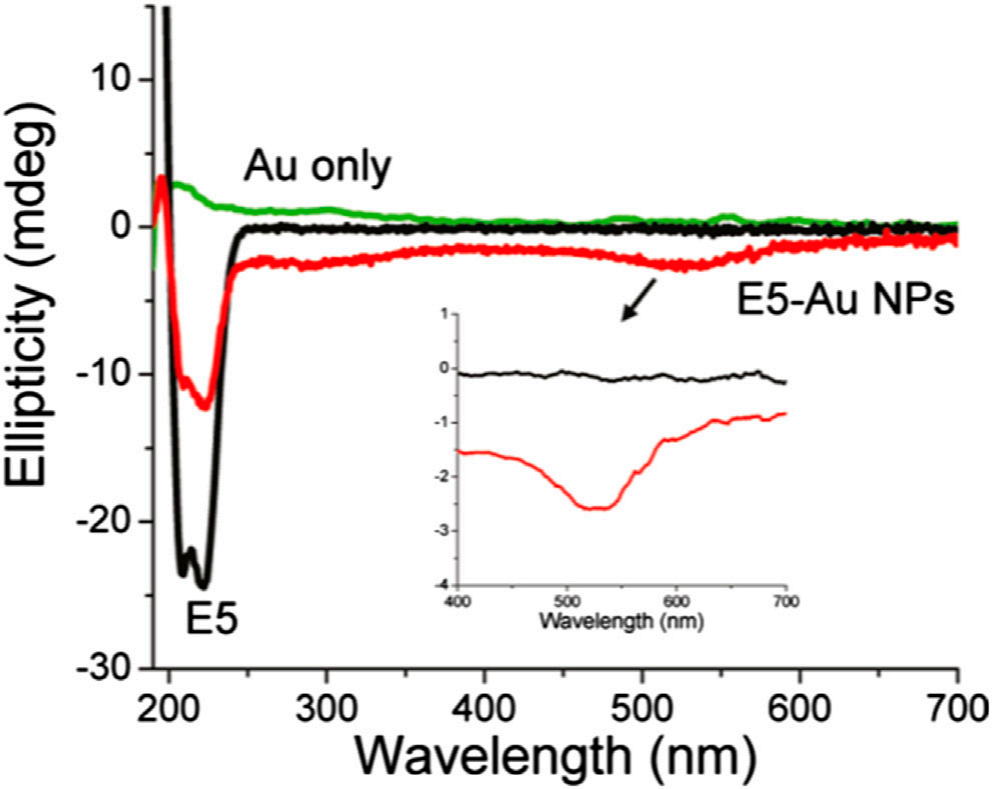
Optical characterization of gold nanoparticles functionalized with the E5 peptide (5.0 μM) with 1.14 _ 1013 particles/ml. CD spectra of E5 peptide gold nanoparticles with or without E5 peptide. The figure and caption have been adapted with permission from Slocik et al., (2011), Copyright ^©^ 2011 American Chemical Society.

Moreover, chiral metallic nanostructures possess plasmonic CD (PCD), a unique chiroptical property due to which the enhanced CD signal can be obtained at the frequency of localized surface plasmon resonance (LSPR) (Fan and Govorov, 2012; Collins et al., 2017; Lee et al., 2017; Zhao et al., 2017; Lu et al., 2018; Mokashi-Punekar et al., 2019; Kim et al., 2020). In 2011, Slocik et al., (2011) fabricated optically active metallic NPs by functionalizing achiral Au NPs with peptides of random coil and *R*-helix secondary structures. The group observed reproducible PCD at ∼520 nm induced by the SPR of the Au NPs for both covalently and non-covalently attached E5 and FlgA3 peptides, respectively (Figure 4). Similarly, Cheng et al., (2017) reported the generation of strong CD signals from plasmonic nanostructures fabricated by grafting spherical Au NPs of different sizes around chiral inorganic silica helical templates. In addition to the inverted CD spectra observed for right- and left-handed helices with sign reversal occurring near the plasmon resonance frequency of the Au nanoparticle, the study also revealed that the amplitude of the CD signal got increased and red shifted with the increase of the diameter of the nanoparticles. Another strategy demonstrated the transfer of chirality of L- or D-cys to cube-shaped Au NPs, resulting in the formation of highly twisted chiral helicoidal structures that displayed strong CD (Lee et al., 2018). Extending this work, the group also studied the evolution of cysteine-induced chirality in film type of assembled helicoid Au NPs by using CD spectroscopy (Lee et al., 2020). Recently, Rajaei et al., (2019) used chiral metasurfaces composed of two-dimensional (2D) planar array of three-dimensional (3D) plasmonic ramp-shaped Au nanostructures to achieve giant CD and dissymmetry factor up to 64% and 1.13, respectively, at the visible range. In another work, Zhang Q. et al. (2019) investigated the origin of PCD possessed by metal NP (Au nanorod)-protein (BSA) complexes at the single-particle level by combining circular differential scattering spectroscopy along with correlated tomographic reconstruction and electromagnetic simulations. The study revealed that the aggregates exhibit strong PCD, while single nanoparticles did not show any detectable chiroptical activity. Apart from Au-based plasmonic nanostructures, an enhancement of two orders of magnitude of the CD signal was observed from a system of l-glutathione(l-GSH)–coated colloidal silver NPs, further attached to bimane chromophore, due the coupling of metal SPR and molecular electronic transition (Lieberman et al., 2008). Moreover, plasmon-induced new CD response was observed at LSPR wavelength range (∼350–500 nm) in case of silver cube-shaped NPs capped with GSH molecules (di Gregorio et al., 2015). Notably, as far as self-assembly–based plasmonic structures are concerned, Au NPs are chemically stable, while Ag NPs possess better optical properties in terms of extinction cross section and near-field enhancement. To exploit their advantages synergistically, Nguyen et al. (Nguyen et al., 2020) synthesized thiolated DNA functionalized gold–silver core–shell nanoparticles that showed significantly enhanced and blue shifted bisignate CD spectra, as compared to the pristine Au counterparts. Certainly, such results obtained from PCD spectroscopy is of utmost importance for the development of next-generation chiral plasmonic devices.

### Vibrational Circular Dichroism (VCD)

As already mentioned, vibrational circular dichroism (VCD) is the differential absorption of RCP and LCP of IR radiation and thus involves the transitions among the vibrational sublevels within the ground electronic state of a chiral substance (Nafie, 2012). Notably, six possible vibrational modes are involved in such transitions (Collins et al., 2017). VCD is relatively young chiroptical technique and was first reported in 1974 by Holzwarth et al. (Holzwarth et al., 1974). It has become one of the most reliable choices for determining the absolute configuration and conformation of chiral molecules due to its inherent advantages, for example, richer spectral information content than ECD spectroscopy, capability of probing molecular interactions and orientation, no requirement of special chromophoric groups, and robust theoretical background (Polyanichko et al., 2011; Petrovic et al., 2015; Kurouski, 2017). A large number of comprehensive reviews are available that highlighted the principle as well as applications of VCD on molecular chirality (Nafie, 2008; Yang and Xu, 2010; Burgueño-Tapia and Joseph-Nathan, 2015; Kurouski, 2017; Nafie, 2018; Krupová et al., 2020).

**FIGURE 5:**
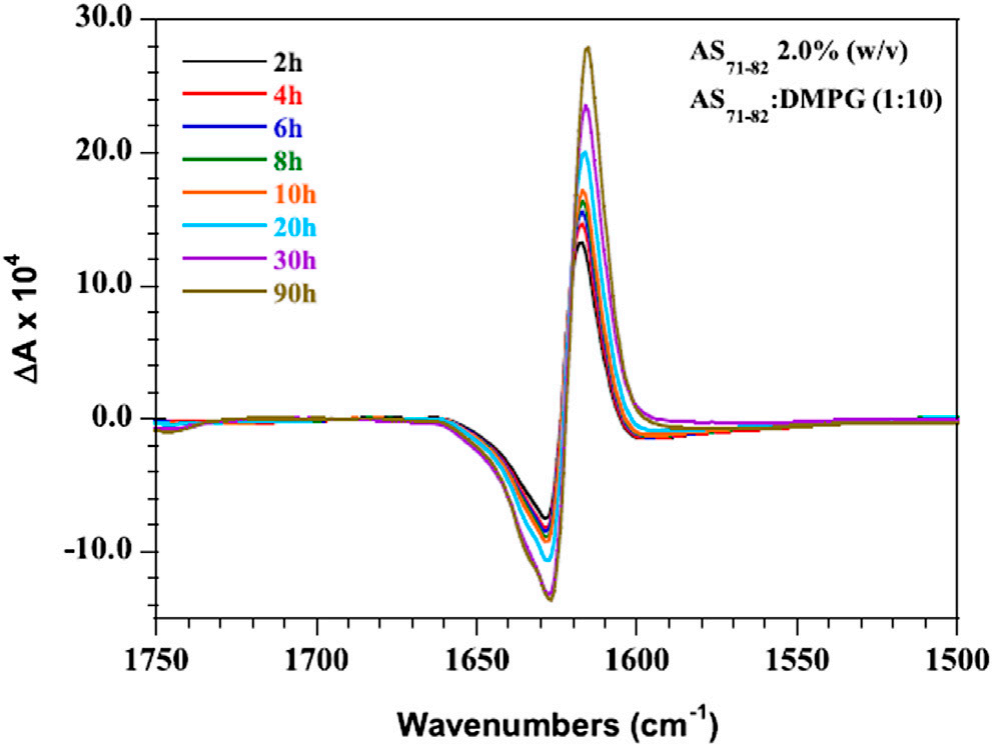
VCD spectra of AS_71−82_ in the presence of DMPG membranes (P:L 1:10, 2.0% AS_71−82_ w/v) recorded as a function of time over a 90 h time period. The figure and caption have been adapted with permission from Martial et al., (2019), Copyright ^©^ 2019 American Chemical Society.

In addition to its widespread use in probing chirality at molecular scale, VCD has also been used to interrogate chirality at membranes, interfaces, and surfaces (Berthier et al., 2002; Novotná and Urbanová, 2012; Das and Pal, 2017). For instance, in a recent work, Novotná et al., (2014) used model membranes of zwitterionic liposomes from 1,2-dimyristoyl-sn-glycero-3-phosphocholine (DMPC) and sphingomyelin (SPM) liposomes to simulate the phospholipid bilayer and nerve cell membranes, respectively. The group further used VCD to investigate the interaction of bilirubin (BR) with DMPC and SPM model membranes as well as polypeptide poly-l-arginine (PLAG) model membrane proteins, as functions of pH and temperature. The study successfully revealed the strong mutual influence between BR and SPM, which may be related to BR neurotoxicity (Novotná and Urbanová, 2015). Similarly, Kocourková et al. (Kocourková et al., 2017) studied the conformation of antimicrobial peptides melectin and antapin in simulated eukaryotic, bacterial, and nerve cell membranes, where the VCD technique was found to be powerful in differentiating *α*-helical and polyproline II-type conformations. In another work, interaction of amyloid polypeptide AS_71−82_ with anionic membranes was investigated by coupling VCD spectroscopy and IR spectroscopy (Martial et al., 2019). As shown in Figure 5, the time-dependent VCD spectra revealed the increase in signal intensity over time, probably indicating the gradual maturation and stabilization of the fibrillar structures. Interestingly, Lizio et al. (Lizio et al., 2018) employed the combination of Raman, ROA, IR, and VCD spectroscopy to elucidate their capability in determining the relative proportion and conformation of *α*-aminoisobutyric acid (Aib) foldamers in membranes as well as in organic solvents. The VCD spectra of L- or D-Phe capped tetrameric Aib foldamers in the membranes of vesicles revealed the retention of the screw-sense preference of an Aib foldamer even after partitioning into a 1,2-dioleoyl-snglycero-3-phosphocholine (DOPC) bilayer. On the other hand, Pazderková et al., (2019) investigated the structural changes of antimicrobial peptide Halictine-1 (HAL-1) due to the interaction with model membranes by combining the use of IR, ECD, VCD, and fluorescence spectroscopies. Here, VCD spectroscopy was specifically used to understand the high concentration–induced structural change of peptides.

Importantly, as far as the experiments to measure VCD spectra are concerned, the major challenge is the relatively weak signals and anisotropy factors. As compared to ECD and IR absorption, VCD signal strength is generally several orders of magnitude smaller (Petrovic et al., 2015; Polavarapu and Santoro, 2020). However, enhanced chiroptical signals can be achieved from high specific surface area material, such as NPs which can be considered as the nanometer-sized analogs of extended flat surfaces (Bieri et al., 2007; Gautier, 2008; Collins et al., 2017; Bhattacharya and Bürgi, 2019). Importantly, in a first-of-its-kind experiment, Gautier et al. (Gautier and Bürgi, 2005) studied the VCD activity of monolayer (N-acetyl-L-cysteine)-protected Au NPs. In another similar work, the group investigated the OA of N-isobutyryl-L-cysteine and N-isobutyryl-D-cysteine molecules adsorbed on the surface of AuNPs by using VCD (Gautier and Bürgi, 2006). Theoretical calculations, conducted in this work, by using density functional theory (DFT) revealed the strong dependence of the VCD spectra on the adsorbed N-isobutyryl-cystine conformation. In order to explain the mechanism of chirogenesis in metal-based transitions, the group proposed the possible induction of chiral “footprint” due to the double interaction of both acid and thiol groups of N-isobutyryl-cysteine with the metal particle surface. Furthermore, mirror image relationship between the VCD spectra of Au NPs modified by both the enantiomers of 1,1’-binaphthalene-2,2’-dithiol (BINAS) was reported in a recent work (Gautier and BüRgi, 2010). The group observed that the VCD spectrum of adsorbed BINAS is similar to the dithiol form, albeit it appears to be different from its free and disulphide form. Recently, VCD spectroscopy was used to study the conformation and configuration of Au_25_(PET)_18−2x_((*R*)-FBINAS)_x_ cluster (PET = 2-phenylethylthiol, (*R*)-FBINAS = (*R*)-5,5′,6,6′,7,7′,8,8′-octafluoro-[1,1′-binaphthalene]-2,2′-dithiol) (Sels et al., 2019). Notably, the study revealed the transfer of chirality of FBINAS to originally achiral PET, which was co-adsorbed within the ligand layer. In another work, Bhattacharyya et al. (Roy Bhattacharya and Bürgi, 2019) successfully obtained the enhanced VCD signal from water soluble gold nanocluster Au_25_(captopril)_18_ when a small amount of cobalt(II) was added in the aqueous solution.

Mostly, VCD measurements involve solution-state samples. Recently, in order to expand its applicability and measure the VCD of different solid samples such as KBr pellets, and films, solid state VCD (SD-VCD) was developed (Sasaki et al., 2013; Mijiddorj et al., 2019; Sato and Kawamura, 2020). In one of the applications, SD-VCD was used to study the mechanism of chiral recognition on a solid clay surface (Sato et al., 2018).

**FIGURE 6:**
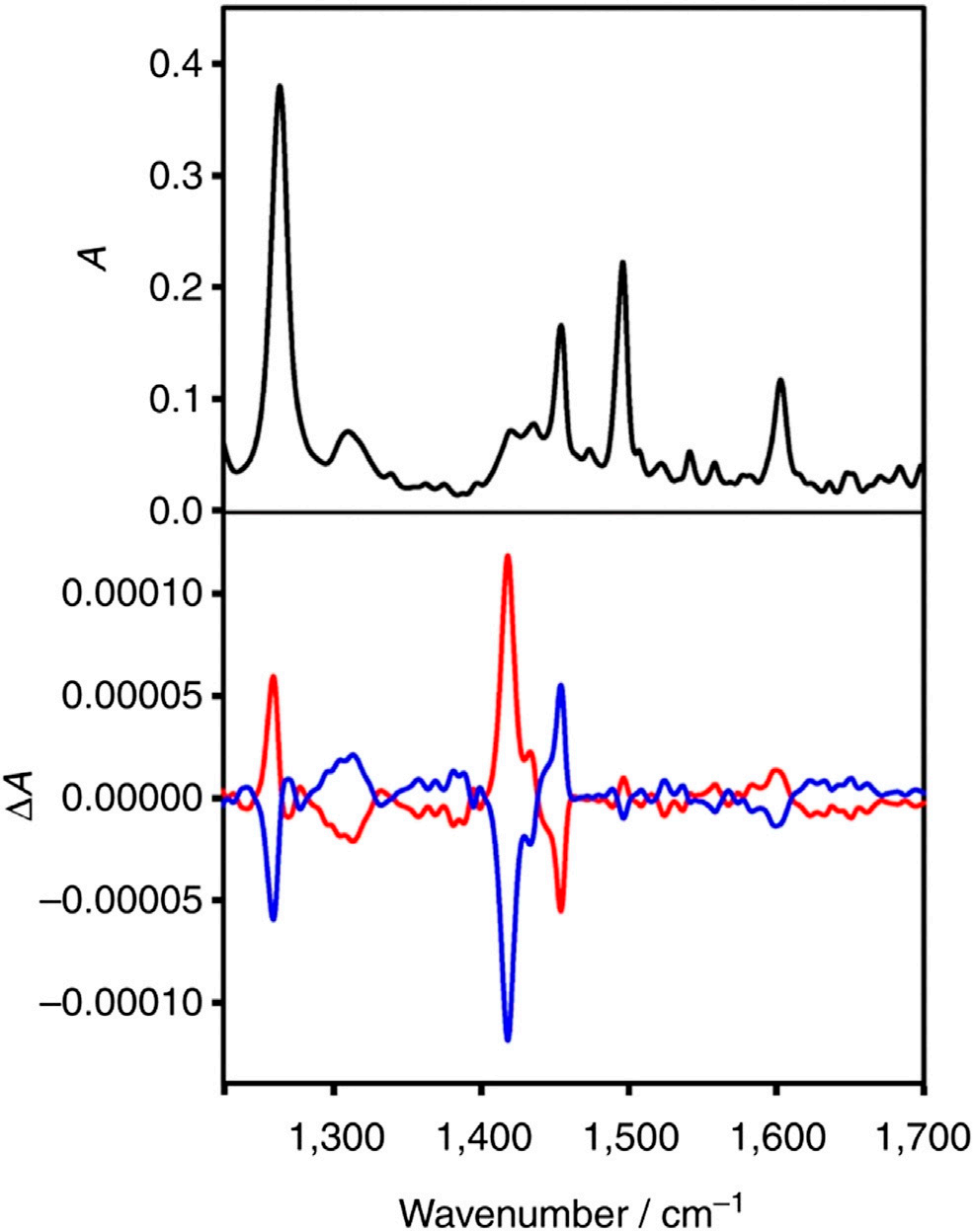
Infrared spectrum of A-Au_38_(2-PET)_24_ (black) and VCD spectra of enantiomer 1 (A-Au_38_(2-PET)_24_, red) and of enantiomer 2 (C-Au_38_(2-PET)_24_, blue). The figure and caption have been adapted with permission from Dolamic et al., (2015), Copyright & 2015 Macmillan Publishers Limited.

In another interesting work, first direct observation of transfer of chirality from chiral metal cluster to achiral molecules adsorbed on its surface was successfully achieved by VCD spectroscopy (Dolamic et al., 2015). The adaptation of chiral conformation by achiral 2-phenylethylthiolate (2-PET) after getting adsorbed on chiral Au cluster was evidenced by the strong VCD signal from the thiolate moiety. Notably the experimental results were further explained by density functional theory (DFT) calculations. Figure 6 shows the VCD spectra of the Au_38_(2-PET)_24_ cluster enantiomers. On the other hand, Yao et al., (2010) investigated the structure of chiral D-/L-penicillamine ligands adsorbed on silver nanoclusters with mean core diameter ∼1.1 nm. The group compared the experimental data with DFT calculations to perform spectral assignments. Accordingly, such studies are important to understand not only the mechanism of chirality transfer but also accurate determination of the configurations and conformations of surface-adsorbed molecules.

### Raman Optical Activity (ROA)

Raman optical activity (ROA) is the complementary chiroptical technique of VCD, capable of providing conformational information even for samples that do not have distinct VCD bands (Barron et al., 2007; Krupová et al., 2020). It is the inelastic counterpart of Rayleigh optical activity (Andrews, 1980) and is essentially measured as the difference between the Raman scattering intensities for right and left circular polarized incident or scattered radiation. Accordingly, there are four kinds of linear ROA corresponding to each of the two circularly polarized states of incident and scattered radiation (Collins et al., 2017; Nafie, 2018; Nafie, 2020). Since its first demonstration in 1973 (Barron et al., 1973), it has already become a matured technique and found widespread applications in diverse fields ranging from chiral molecules to viruses (Blanch et al., 2002; Blanch et al., 2003; Zhu et al., 2005; Hoffmann, 2012; Parchaňský et al., 2014; Haraguchi et al., 2015; Gąsior-Głogowska et al., 2016). Notably, ROA is often measured in terms of the dimensionless circular intensity difference (Hoffmann, 2012).

Besides, ROA has also been used in the study of surface chirality. In one of such pioneering works, Hecht and Barron, (1994) worked out the detailed mechanism of incoherent Rayleigh and Raman optical activity from chiral surfaces and predicted that the associated circular and linear polarization optical activity observables could be three orders of magnitude larger than those from bulk samples. The group successfully used differential decomposition of the normal incidence transmission Mueller matrix to determine circular birefringence and dichroism values. In another work, birefringence-induced ROA in uniaxial crystals with rutile structure was demonstrated by slightly misaligning the incident light with respect to the crystal *c*-axis direction (Hoffman et al., 1994). Recently, scattered circular polarization ROA strategy was used to study the chirality of chiral two-dimensional nanostructured ZnO films and chiral nanostructured ZnO powders (CNZPs) (Duan et al., 2015a). Particularly, the observed ROA revealed the highly crystalline nature of CNZPs.

At this point, it is noteworthy to mention that despite having excellent capability in extracting the absolute configuration and conformation, ROA suffers from weak signal intensities of about 3–5 orders of magnitude smaller than the original Raman scattering intensity, which is already about six orders of magnitude weaker than the incident beam. Obviously, such limitations of spontaneous ROA warrant data acquisition time and higher sample concentration requirement (Abdali and Blanch, 2008; Pour et al., 2018; Krupová et al., 2020). Nevertheless, the advantages of surface-enhanced Raman scattering (SERS) and resonance Raman scattering (RRS) can be utilized to enhance the corresponding ROA intensity (Johannessen et al., 2007; Nagy et al., 2016; Hu et al., 2018). Recently, resonance ROA was utilized to study the enantio–enriched (6,5) single-walled carbon nanotubes (SWNTs) sorted by density gradient ultracentrifugation (Magg et al., 2016).

**FIGURE 7:**
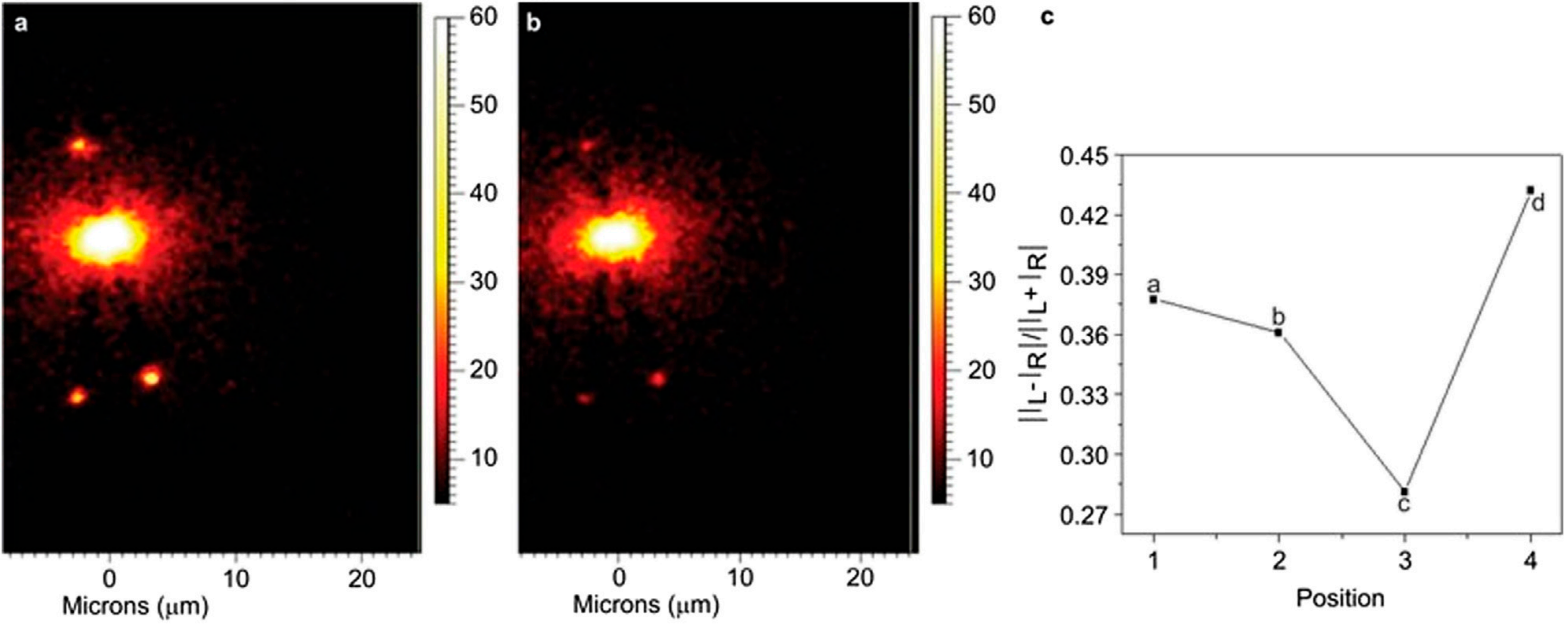
Imaging of the Raman peak of FGGO at 1593 cm^−1^, excited by circular polarization, and the fitted CIDs at four different points. **(A)** Results for left circular polarization. **(B)** Results for right circular polarization. **(C)** The fitted CIDs at four points of the nanostructure. CID, circular intensity difference; FGGO, fmoc-glycyl-glycine-OH. The figure has been adapted with permission from Sun et al., (2013), 2013 CIOMP.

Notably, the combination of SERS and ROA was first considered almost three decades ago by Efrima, (1983) who predicted that enhanced ROA signals from chiral molecules adsorbed onto metal surfaces can be obtained due to the large electric field gradient that exists near the surface. Efrima also developed the first theoretical framework for explaining the enhancement of ROA of chiral molecules adsorbed on metal surfaces (Efrima, 1985). In spite of these pioneering works and following theoretical developments, experimental developments for surface-enhanced ROA (SEROA) did not gain sufficient pace during the last decades, mainly because of the difficulties (e.g., large signal-to-noise ratio and complicated spectra) associated with the experiments (Janesko and Scuseria, 2009). However, significant number of efforts have been made in the recent years to revitalize the application of SEROA (Janesko and Scuseria, 2006;^2009^;Pour et al., 2011;Ren et al., 2017). For example, Kneipp et al., (2006) successfully obtained stereochemical information of adenine adsorbed on Ag nanoparticles by measuring the SEROA at significantly reduced excitation power, shorter data acquisition time, and lower molecular concentration as compared to classical ROA measurements. In another exciting work, Sun et al., (2013) reported an alternate method of achieving SEROA which can be useful in reducing the artifacts present in SEROA spectra due to the influence of chemical enhancements. The method involved the remote excitation of ROA of chiral fmoc-glycyl-glycine-OH (FGGO) molecules by using chiral Ag nanowires as plasmonic waveguides. The applicability of the method was demonstrated by obtaining ROA image and measuring the ROA spectra as well as circular intensity difference (CID), as shown in Figure 7. Similarly, remote-excited ROA of adenine was realized by surface plasmon propagating along Ag nanowires (Xia et al., 2014). On the other hand, surface-enhanced resonance ROA (SERROA), that involves the coupling of the resonance Raman scattering with surface plasmon enhancement, has also been reported (Abdali et al., 2007; Johannessen et al., 2007). However, SERROA can only be achieved only when there is an overlap between the electronic transition and plasmon resonance frequencies.

### Nonlinear Optics on Surface Chirality

Both SHG and SFG are established spectroscopic techniques specifically used to study surface chirogenesis. In theory, these two nonlinear optical processes can be uniquely described by the second-order susceptibility, *χ* ^(2)^, tensor (referred to as *χ* ^(2)^ tensor hereafter) under the point symmetry consideration of C_∞_ when chiral molecules are adsorbed at surface/interface (Maki et al., 1995; Kriech and Conboy, 2003; Mitchell, 2006). Sometimes, the adsorbed molecules should have a certain degree of orientation in order to enhance the C-SHG and C-SFG signal, and thereby, they are structural effects of molecules in response to a polarized field (Simpson, 2002; Mitchell et al., 2005). To derive the nonvanishing *χ* ^(2)^ tensor elements related to surface chirality, *χ* ^(2)^ tensor transformation from molecular frame to laboratory frame using the Euler rotation matrix is required, where *χ*_xyz_ refers to the fact that the direction of the second-order nonlinear polarization flipped (rotated) from the plane of fundamental polarization indicates the chiral-specific component (Petralli-Mallow et al., 1993). Regarding the polarization settings in experiments, the polarization combinations of psp, spp, and pps are responsible for C-SHG and C-SFG, while ssp, sps, pss, and ppp are in charge of achiral contributions at the surface/interface. This section will summarize the arrangement of measurement setup and selected applications of C-SHG and C-SFG in investigating surface chirality.

### Surface Chirality Measured by Second-Harmonic Generation (SHG)

The scheme for polarimetric measurements of C-SHG can be divided into two main categories: linear polarization to planar surface and circular polarization to planar surface, leading to the optical activities of SHG-CD, SHG-LD, and SHG-ORD. In SHG-CD, first demonstrated by Hicks and coworkers (Petralli-Mallow et al., 1993), chirality results in the SHG intensity difference between the interactions of RCP and LCP, and the signal contrast ratio can be on the order of unity which is more than two orders of magnitude stronger than linear CD (Petralli-Mallow et al., 1993; Hicks et al., 1994). Notably, the sign of SHG-CD ratio (also known as degree of chiral excess, DCE, or enantiomeric excess) indicates the handedness of chiral molecules. Such circular-difference effect is derived from the interference between chiral and achiral components of the *χ* ^(2)^ tensor (Byers and Hicks, 1994; Fischer and Hache, 2005; Rodriguez et al., 2007). Because the *χ* ^(2)^ tensor components, oriented normally to the surface, contribute to surface SHG, the arrangement of measurement setup uses a collimated (or weekly focused) laser beam with an angle of incidence (typically 45^o^) relative to the surface normal to probe the chiroptical effects. Then, the reflected and/or transmitted SHG emitted from the surface is measured. In general, SHG-CD is incorporated with a spectroscopy to have a full view of the resonance effect of the adsorbed molecules (Corn and Higgins, 1994). In SHG-LD, the SHG polarization–dependency curve is measured by rotating the incoming linear polarizations from 0^o^ to 360^o^ using a half-wave plate, with an angle of incidence (typically 45^o^) relative to the surface normal. The reflected and/or transmitted SHG is detected at s- or p-polarization. The linear-difference effect can be found by comparing the signal at 45^o^ and −45^o^ (or 135^o^) of the p-direction of the incident beam (Fujiwara et al., 2004; Xu et al., 2009; Lin et al., 2014). Similar to SHG-CD, the sign of SHG-LD ratio also reflects the handedness of chiral molecules. In SHG-ORD, an oblique incidence of the p-polarized laser beam is used to probe surface/interface, and the direction of SHG polarization is measured with an analyzer to determine the rotation angle in SHG. Similar to SHG-CD, a spectrally resolved measurement is performed to better understand the resonance effect of molecules (Byers et al., 1994a; Hache et al., 2003). In the following, we will lay emphasis on illustrating the selected works of SHG-CD and SHG-LD on surface chirality.

**FIGURE 8:**
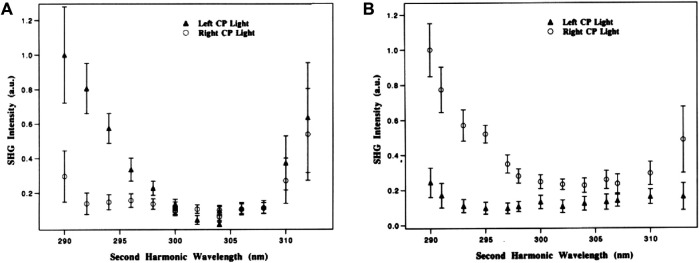
**(A)** Relative SHG efficiencies as the laser frequency is varied, plotted as a function of the second-harmonic wavelength for the *R* enantiomer of BN using left circularly polarized (▲) and right circularly polarized (○) light. Intensity from neat water: 0.01 au. **(B)** Relative SHG efficiencies as the laser frequency is varied, plotted as a function of the second-harmonic wavelength for the *S* enantiomer of BN using left circularly polarized (▲) and right circularly polarized (○) light. Intensity from neat water: 0.05 au. The figure and caption have been adapted with permission from Petralli-Mallow et al., (1993), Copyright ^©^ 1993 American Chemical Society.

In 1993, a pioneering work conducted by Petralli-Mallow et al. (Petralli-Mallow et al., 1993) successfully revealed the OA effect of 1,1’-binaphthalene-2,2’-diol (BN) at an air/water interface by using SHG-CD spectroscopy. The group observed that the chiroptical response from (*R*)- and (*S*)-BN depends on the helicity of incident polarized light, giving rise to opposite CD effects by the two BN enantiomers, as shown in Figure 8. Importantly, due to SHG-CD being electric dipole allowed, it is three orders of magnitude stronger than that measured by linear CD spectroscopy. Because SHG intensity is the square of the *χ* ^(2)^ tensor, this further enhances the difference effect. Moreover, it is observed that without aggregate formation of molecules, a preferred orientation of adsorbed molecules at the surface is hypothesized to be the main contributor to amplify SHG-CD. In 1994, Verbiest et al. (Kauranen et al., 1994) used a Langmuir–Blodgett (LB) film embedded with bacteriorhodopsin molecules for studying surface chirality. The adsorbed molecules present a local ordering in a lattice and randomly oriented on the length scale smaller than an optical wavelength. In the measurement, the incoming polarization on the sample was modulated by a quarter-wave plate (Maki et al., 1997) to continuously change the polarization state from left-circular to linear to right-circular, and subsequently, the surface SHG is recorded at s- and p-polarization in transmission or reflection. Importantly, a theory that includes electric and magnetic dipole contributions to C-SHG was proposed to explain the experimental results. Following this method and accompanying theory, Kauranen et al. (Kauranen et al., 1994; Maki et al., 1995) further included the nonlinear Fresnel factors to accurately describe the C-SHG, and a magnetic dipole contribution ∼10% of the electric dipole one was found on the LB monolayers of a synthetic chiral polymer, chromophore functionalized poly(isocyanide). Similarly, Rodriguez et al., (2007) observed OA in thin films of a chiral poly(3-alkylthiophene). In the experiment, the dependency of SH intensity on the rotation angle of the half-wave plate (combined with a quarter-wave plate at fixed angle placed behind it) at a specific laser incidence angle was resolved into s- and p-polarization in transmission and reflection. Notably, a deeper analysis of the susceptibility tensor elements (Rodriguez and Sourisseau, 2002) determined in this work suggested that, analogous to linear CD, the possible origin of the observed chiroptical response could be the interference between electric and magnetic dipole contributions.

**FIGURE 9:**
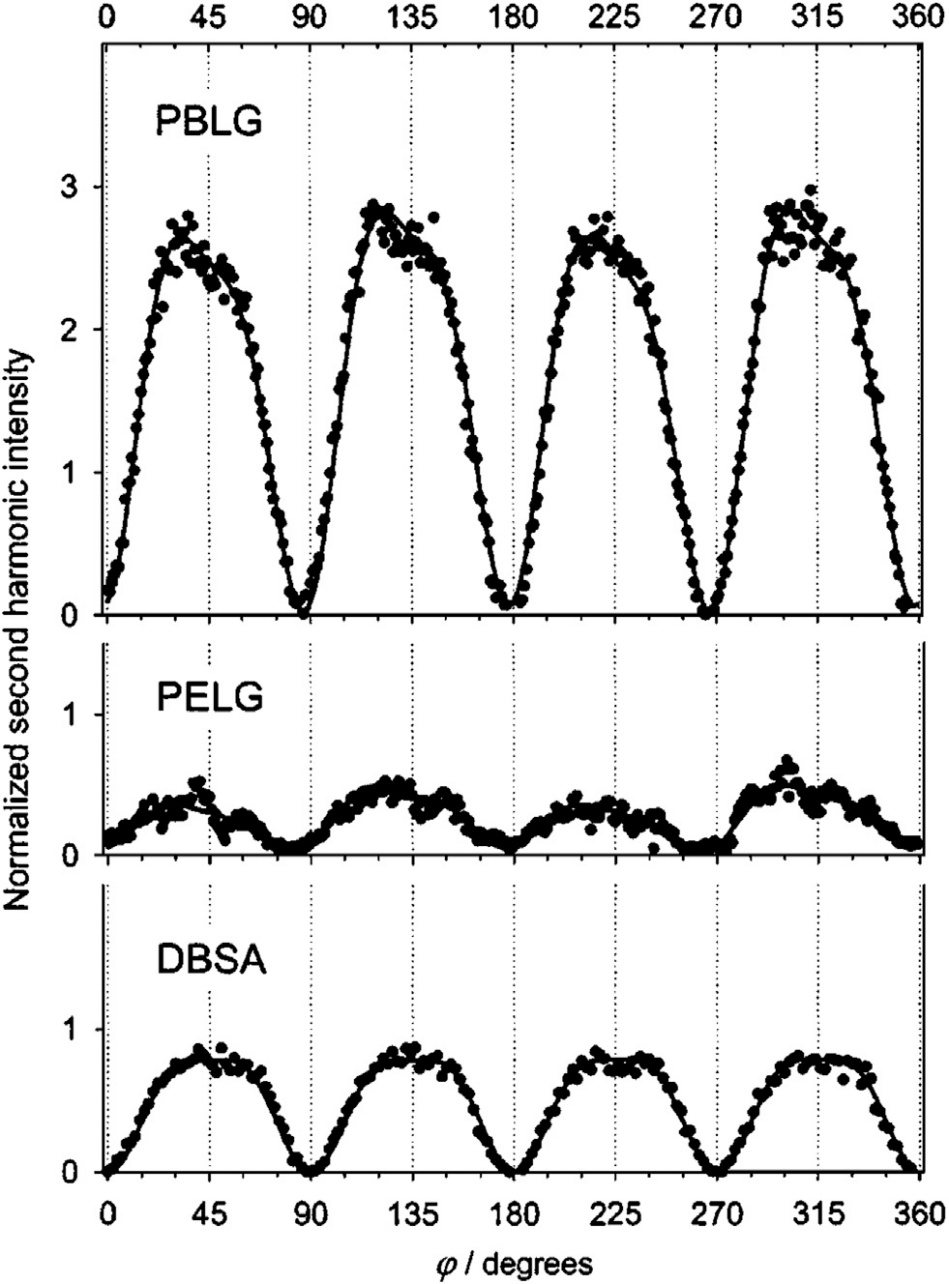
Rotation traces I_2*ω*_(*φ*) for the polypeptides PBLG and PELG and for the achiral surfactant DBSA adsorbed at the air/water interface. The solid lines are fits of the data to Eq. (8). Note that the traces for the chiral interfaces PBLG and PELG are asymmetric about *φ* = 180°, and the trace for the achiral interface DBSA is symmetric. The figure and caption have been adapted with permission from Mitchell et al., (2005), Copyright ^©^ 2005 AIP Publishing.

Regarding the issue of orientation or structure of molecules on surface, Byers et al. (Byers et al., 1994b) investigated a monolayer of oriented chiral molecules (BN) and found that SHG-CD not only depends on the handedness of incident polarized light but also on the adorbed molecules pointing upward or downward from the surface, presenting a positive or negative value of SHG-CD, respectively. Afterward, Sioncke et al., (2001) investigated a chiral, anisotropic LB film of helicenebisquinone, in order to separate the effect of SHG-CD from anisotropy. Results showed that SHG-CD is independent of molecular orientation when sample is rotated, and conversely, the anisotropy depends on the azimuthal angle of the sample, leading to a CD effect. Furthermore, Mitchell et al. observed the OA response from a polypeptide *α*-helix adsorbed at the air/water interface (Mitchell et al., 2005). In this experiment, poly-(*γ*-benzyl-L-glutamate) (PBLG) and poly-(*γ*-ethyl-L-glutamate) (PELG) were examined, which assisted to form an *α*-helix of the polypeptide chain. And the effective susceptibilities for C-SHG can be resolved by p- or s-polarization detection while the incident polarization state was continuously varied using a quarter-wave plate, as shown in Figure 9. Result showed that a symmetric or asymmetric SHG polarization dependency represents an achiral or chiral surface, respectively. The most important finding was that a structural effect from the molecular tilting of the individual amide chromophoric subunits with respect to the principal axis of the helix is responsible for surface chirality. Furthermore, Mitchell studied the SHG-OA effect of tryptophan derivative Nα-(*tert*-butoxycarbonyl)tryptophan (Boc-Trp) at an air/water interface (Mitchell, 2006). The *χ* ^(2)^ tensor of chiral and achiral monolayers were determined both experimentally (under two-photon resonant and nonresonant excitation conditions) and theoretically (with ZINDO/S), which agreed quite well with each other. Based on these results, it is concluded that the surface chirality is from a structure effect of the absolute orientation (tilt angle *θ* and twist angle *ψ*) of the long axis of indole (polar and achiral) chromophores organized in chiral assemblies at the interface. Moreover, Zou et al., (2006) studied the chirality at polymerized 10,12-tricosadynoic acid (PTDA) LB films on quartz substrate using SHG-CD spectroscopy and polarized angle dependence measurements. It was found that PTDA films showed surface chirality when Cu(II) ions appeared in the subphase solution but did not show surface chirality when the subphase solution contains Cd(II) ions or is pure water. This phenomenon is probably from the metal ions in the subphase solution, which induce a conformational change of the polydiacetylene chain in PTDA films. In addition, the Cotton effect was also observed in the SHG-CD spectra when PTDA films are deposited from Cu(NO_3_)_2_ subphase, further demonstrating that surface chirality can be formed by a conformational change of achiral molecules.

On the other hand, Fujiwara et al. first studied the chiral molecular aggregates at a liquid/liquid interface by SHG-CD spectroscopy (Fujiwara et al., 2004). The SHG-CD spectra are similar to that measured by linear CD spectroscopy of J-aggregates in bulk solution. One difference from linear CD is that the zero difference effect is not at the absorption maximum of molecules, which may ascribe to the twisted configuration of H_4_TPPS^2-^ in J-aggregates. In addition, the asymmetric chiral structure of J-aggregates has been proven to enhance the SHG signal, and the surface chirality can be contributed from the magnetic dipole interactions. Furthermore, in a totally internal reflection (counter-propagating) mode of SHG-CD spectroscopy (Tokunaga et al., 2009), an objective lens was used to shift the position of the incident beam to one side from the lens center and collected the SHG signal at another side. The system was used to discriminate the chirality of molecular aggregates of porphyrin on a glass plate. It is found that the SHG-CD spectra of bilirubin-BSA complex are different from that of racemic bilirubin, manifesting that an M-conformation is appeared in the bilirubin-BSA complex. An advantage of this setup is the easy integration into an SHG-CD microscopy to image surfaces/interfaces and cell membranes. Regarding the protein-membrane interactions, Verbiest et al. (Hicks and Petralli-Mallow, 1999) used an electron transfer protein, cytochrome *c*, to adsorb on self-assembled mono- and bilayers at the solid/liquid interface in order to study the oxidation/reduction state of the protein. It is observed that due to the electrostatic interactions of zwitterions and a net negative charge from the lipid surface, the protein is tightly bonded not reducible by adding ascorbic acid. Afterward, the same group followed the identical experimental conditions and found that the cytochrome *c* protein is reducible by ascorbic acid when adsorbed at alkanethiol self-assembled monolayers but is not reducible at phospholipid/alkanethiol hybrid bilayer membranes (Petralli-Mallow et al., 2000). The phenomenon reveals that cytochrome *c* is reducible by ascorbic acid for negatively charged or hydrophobic monolayers, but not for negatively charged phospholipid and zwitterionic bilayers. This work demonstrates the capability of SHG-CD in monitoring protein-membrane dynamics.

**FIGURE 10:**
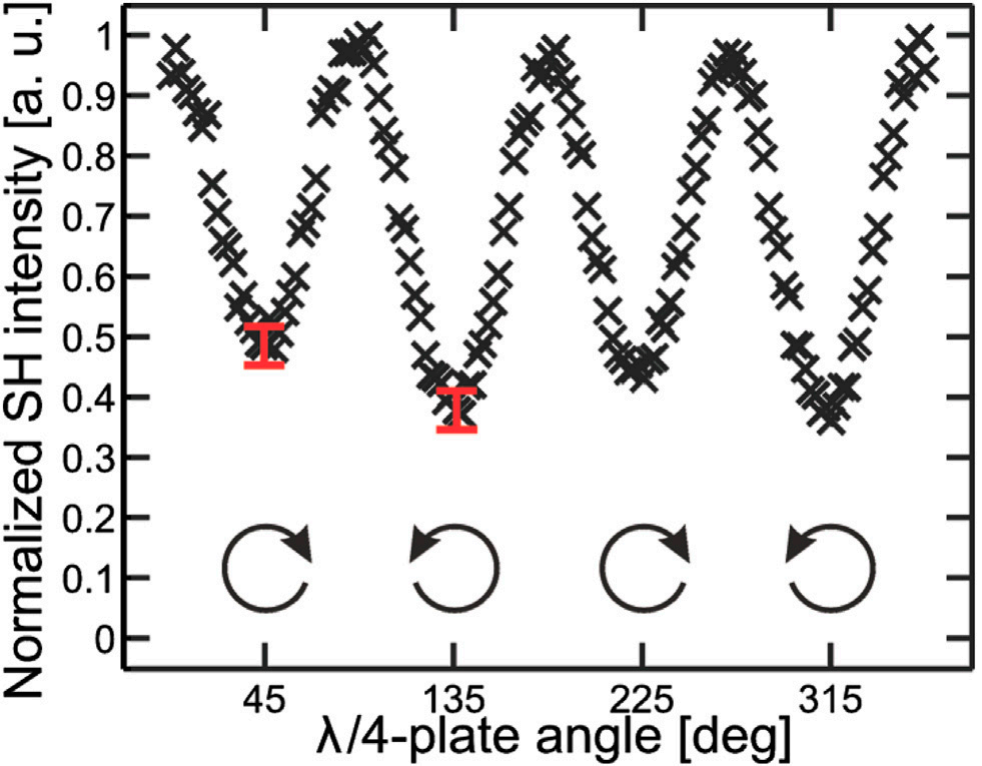
Measured SHG response from a chiral thin film of THBQ as a function of the rotation angle of the QWP. The angles 45^o^, 135^o^, 225^o^, and 315^o^ correspond to CP with varying handedness. The measured SHG-PD response was 0.24. The figure and caption have been adapted with permission from Huttunen et al., (2010), Copyright ^©^ 2010 American Chemical Society.

Later on, Huttunen et al., (2009) conducted a simulation work for a different measurement scheme that used a focused circularly polarized laser beam at normal incidence to the surface, holding an azimuthal symmetry about the direction of beam propagation. Notably, this strategy can be used to prevent the interference of SHG by in-plane anisotropy that would produce a chiroptical response commonly shown in traditional SHG-CD measurements. Therefore, such measurement is only sensitive to surface chirality isolated from any anisotropy effect of molecules. Another advantage is that it is adaptable to microscopy techniques on surfaces/interfaces due to the normal incidence of the laser beam. Additionally, in the plane wave approximation, the incoming laser beam with an angle between the surface normal can couple the surface nonlinearities offered by the *χ* ^(2)^ tensor components oriented normally to the surface. Because a circular polarization reveals full vectorial properties within the focal volume, the C-SHG signal is undoubtedly produced from the direction of surface normal. The same group continued to confirm and validate this work using two LB films, one of which was chiral and anisotropic, while the other was achiral and isotropic (Huttunen et al., 2010). The first LB film was fabricated from chiral molecules, tetradodecyloxyhelicenebisquinones (THBQs), that belong to a C_2_ point symmetry, while the second film was fabricated from 2-docosy-lamino-5-nitropyridine (DCANP) molecules with a C_1h_ point symmetry. To confirm the molecular orientation that would make a false interpreting of C-SHG, the two films were measured at different relative directions between the anisotropic axis of the sample and the incident polarization direction. Results showed that there is no SHG-CD response in case of the DCANP film. However, an average SHG-CD value of 0.24 was measured in the THBQs film (Figure 10), confirming the insensitivity of this technique to in-plane anisotropy. Beside the above applications, SHG-CD has been used to study chirality at the air/water interfaces of aqueous (*tert*-butoxycarbonyl)-tryptophan-tryptophan (Boc-Trp-Trp), *R*- or *S*-citronelloxy-cyanobiphenyl monolayer (Crawford et al., 1994; Manaka et al., 2005), anisotropic achiral oxidized Si (001) film (Li et al., 2006), glass substrate of *G*-shaped nanostructures (Mamonov et al., 2014), etc. In recent years, by combing the surface specificity and optical sectioning of SHG, SHG-CD has been integrated into nonlinear optical microscopy to image collagenous tissues (Zhuo et al., 2017a; Zhuo et al., 2017b; Schmeltz et al., 2019; Schmeltz et al., 2020). The information of *χ* ^(2)^ tensor, molecular orientation, polarity, and chirality is extracted with delicate polarimetric measurements and tensor analyses. The corresponding results can be presented in terms of images.

Although the conventional SHG-CD spectroscopy can reveal the chiral information at the surface in a label-free and noninvasive manner, the typical shortcomings are the requirement of a large phase difference between chiral and achiral terms in the *χ* ^(2)^ tensor and the difficulties in implementing a spectrally resolved measurement. In this context, SHG linear difference (SHG-LD) technique has been successful in investigating surface chirality with rotated linear polarizations to obtain a polarization dependency of SHG. In 1995, Verbiest et al. (Verbiest et al., 1995) first used SHG-LD to study LB monolayers of the *S*-enantiomer of a poly(isocyanide) polymer. The measured SHG from isotropic chiral surfaces is different for the linearly polarized excitation at 45° and -45° relative to the p-polarized direction. The difference effect is of the same order of magnitude with SHG-CD but is of orders of magnitude larger than that measured by linear CD. In contrast to SHG-CD, SHG-LD does not require the phase difference between the chiral and achiral susceptibility terms, thereby allowing it to be observed under nonresonant excitation. In general, SHG-LD serves as a partner technique to SHG-CD, both used for studying surface chirality. Some representative works of SHG-LD on surface chirality have been illustrated below.

**FIGURE 11:**
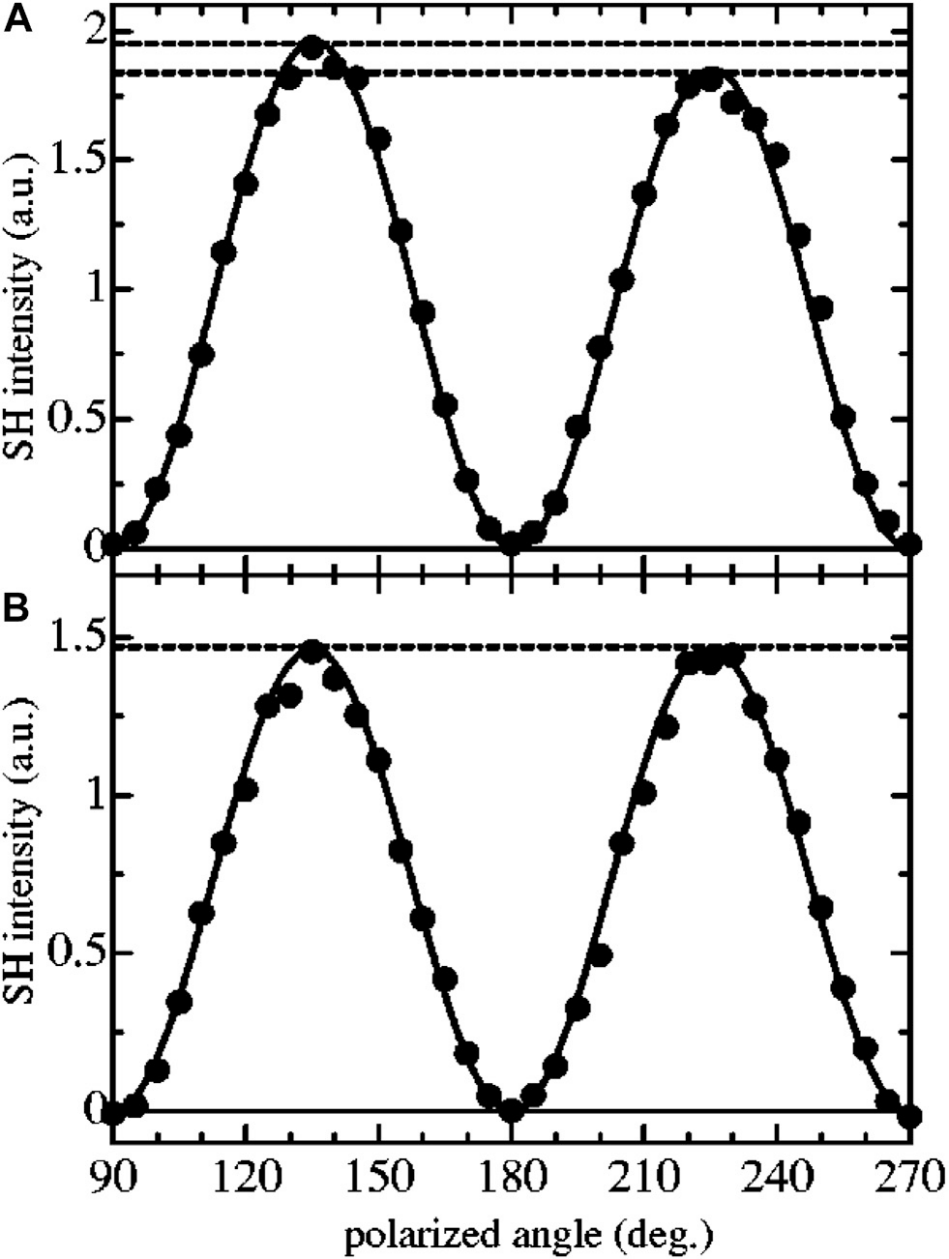
Experimental results for the polarized angle dependence measurement for **(A)** 6OCB and **(B)** *S*-CCB monolayer. SH light was detected for the transmitted direction. In this method, the analyzer angle was fixed and the SH signals were measured as changing the input polarized angle. Solid circles represent the experimental results, and the solid line shows the fitting curves using the appropriate formula *I*(2*ω*)_s_ ∝ |*s*_15_ sin2*γ*_*i*_ - *s*_14_ cos^2^ *γ*_*i*_|^2^. The figure and caption have been adapted with permission from Fujimaki et al., (2003), Copyright ^©^ 2003 AIP Publishing.

Fujimaki et al., (2003) have used SHG-LD combined with Maxwell’s displacement current measurement to investigate the air/water interface of chiral (*S*)-4’-citronelloxy-[1,1’-biphenyl]-4-carbonitrile (*S*-CCB) and achiral 4’-hexyloxy-[1,1’-biphenyl]-4-carbonitrile (6OCB) monolayers. SHG-LD was used to measure the chirality and molecular orientation at surface, while the measurement of Maxwell’s displacement current was used to detect the phase transition in monolayers. With the above two measurements, the molecular motion of monolayers on the water surface was monitored and analyzed during compression. Importantly, the hyperpolarizability *β*_123_ was proven to strongly depend on the molecular chirality, and the ratio of *χ* ^(2)^ tensor elements, *S*_14_/*S*_15_, indicated the degree of monolayer chirality. Result showed that an *S*_14_/*S*_15_ ratio of ≈1/30 was obtained for the *S*-CCB monolayer according to the polarized angle dependence measurement at s-output polarization, as shown in Figure 11. However, the measured ratio was very small for 6OCB monolayer. This result corresponds well with the predication of *β*_123_/*β*_333_ using quantum chemical calculations. Then, Fujiwara et al., (2004) measured a characteristic OA effect from the J-aggregates of achiral porphyrin molecules at heptane/water interface by SHG-LD. In this work, the measurement of SHG polarization dependency was performed by rotating a half-wave plate at the molecular resonance wavelength for excitation. The SHG intensity difference was clearly found between the rotation (polarization) angles at +45^o^ and 45^o^ (referring to p- and s-polarization, respectively). The surface chirality is originated from a helical alignment of the J-aggregates from achiral H_4_TPPS^2-^ ion-associated with cetyltrimethylammonium (CTA^+^), which causes a magnetic dipole effect. Following the work of Zou et al., (2006), SHG-LD measurement with s-polarization detection was used to verify the chirality of monolayers, in which an intensity difference was shown at the 45^o^ and 135^o^ polarized angles in the dependency curve. Through the curve fitting under C_∞_ symmetry assumption, the degree of the CD effect, represented by *S*_14_/*S*_15_, was determined for polymerized TDA monolayers deposited from different solutions. It is interesting to note that surface chirality can be formed with a conformational change of achiral molecules caused by metal ions in the subphase solution.

Furthermore, Xu et al., (2009) used SHG-LD to measure the chirality of Langmuir monolayers formed with achiral 5-octadecyloxy-2-(2-pyridylazo)phenol (PARC18) molecules at the air/water interface. Based on the SHG polarization dependency curve measured at s-polarization, the DCE of the Langmuir monolayer was determined according to the SHG intensity difference between the polarization angles at 45^o^ and 135^o^. Results showed that the chirality of the monolayer is correlated with the relative concentration of the two opposite chiral states at different experimental conditions and is mainly from the spontaneous and inhomogeneous aggregation of molecules resulted in symmetry breaking (supramolecular chirality) rather than the compression-induced effects. This study is consistent with Fujiwara et al., (2004) that the surface chirality can be formed by the aggregation of achiral molecules. Following the previous application, Lin et al., (2014) studied the surface chirality of Langmuir monolayers of achiral porphyrins at the air/aqueous interface, which involved the mechanism to form supramolecular chirality with two metal irons, Zn^2+^ and Cu^2+^, in the subphase solution. The Langmuir monolayers of a tetraphenylporphyrin derivative (TPPA2a), which has two long alkyl (hydrophobic) chains and two ester groups, and a TPPA4 (an analog of TPPA2a but without ester groups) were used to confirm the coordination mechanism. In the SHG-LD result, it is concluded that the chirality is originated from the twisted packing of TPPA2a molecules and Cu^2+^ would change the handedness of the TPPA2a helix. In addition, Zn^2+^ forbids the formation of supramolecular chirality of porphyrin assemblies in contrast to Cu^2+^ that facilitates the chiral formation. And the metal irons have a negative effect to form supramolecular chirality when interact with the central nitrogen of porphyrin rings but have a positive effect when interact with the oxygen atoms of the ester groups. Later on, Lv et al., (2015) performed *in situ* measurements of monolayer chirality at the air/water interface with the C-SHG signal amplified by the amphiphile/tetrakis(4-sulfonatophenyl) porphyrin (TPPS) J-aggregates (supramolecular chirality). Based on the SHG-LD results, the chiral amphiphile-tyrosine derivative (TyrC18) monolayers on water and TPPS solution subphase showed no detectable SHG-LD signals, respectively. Furthermore, the measured DCE ratio was up to 200%, and the absolute chiral configuration can be differentiated by the sign of DCE. It is concluded that the amplification of C-SHG is attributed to asymmetrically molecular orientation and increased hyperpolarizability of the J-aggregates benefited from TyrC18.

**FIGURE 12:**
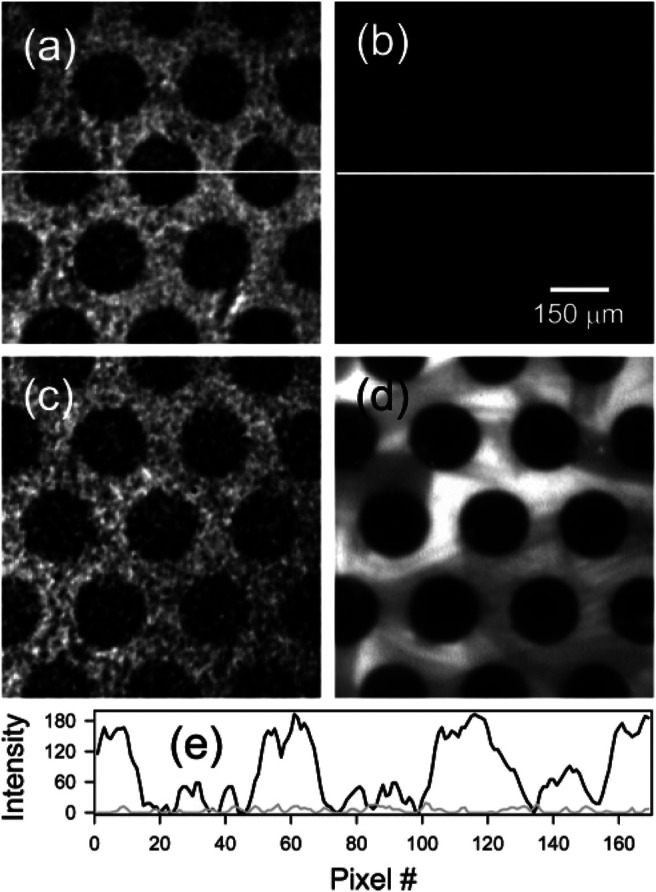
*μ*CP POPC PSLB imaged with C-SHG using **(A)** RBN, **(B)** a racemic mixture of RBN and SBN, and **(C)** SBN. A different bilayer was also imaged with Rh-DOPE using fluorescence microscopy **(D)**. Line scans through the center of image a (black) and b (gray) are shown in part **(E)**. The figure and caption have been adapted with permission from Kriech and Conboy, (2005a), Copyright ^©^ 2005 American Chemical Society.

A different measurement scheme of SHG-LD is proposed by Matthew et al. (Kriech and Conboy, 2003), who implemented SHG-LD on a setup of counter-propagating beam geometry to study the binding affinity of an amphipathic helical peptide, melittin, to a planar supported lipid bilayer (PSLB) of 2-oleoyl-1-palmitoyl-*sn-glycero*-3-phosphocholine (POPC). This approach is able to isolate C-SHG from those achiral SHG contributions by carefully choosing the input and output polarizations. Due to the measurement geometry, the SHG is coherently built along the direction of surface normal (z-direction), and the SHG of x-polarization corresponds to the magnitude of surface chirality. Results showed that melittin experienced a structural change that formed a well-ordered secondary structure, *α*-helix, with a net orientation along the surface normal when intercalating into a lipid membrane, as compared to a random coil in solution. Here, the SHG signal was measured at x-polarization while the input polarization was continuously changed from 0^o^ to 360^o^. The same group continued to develop a compact theory to elaborate this counter-propagating detection system (Kriech and Conboy, 2004) and used it to measure the kinetics of *R*- and *S*-1,1’-dinaphthalene-2,2’-diol (RBN and SBN) binding to a POPC bilayer. The enantiomeric excess of RBN and SBN in lipid membranes were also measured, which demonstrates that the two enantiomers undergo complete exchange in the membrane, and the surface enantiomeric excess depends on the enantiomeric composition in the solution (Kriech and Conboy, 2005b). As a novel application of SHG-LD, the same group imaged surface chirality for the first time using the extended form of SHG microscopy, as shown in Figure 12 (Kriech and Conboy, 2005a). In this experiment, the discrimination between the symmetry of RBN and SBN at a PSLB of POPC was achieved, which further provides the spatial distribution information of absorbed molecules on a bilayer. Importantly, without the need for the phase information of *χ* ^(2)^ tensor elements, *χ*_xyz_ can be uniquely obtained using the polarization combination of P_in_/S_out_. Therefore, by carefully choosing the used polarization setting for the counter-propagating SHG-LD measurement, the chiral and achiral tensor elements at surfaces can be easily isolated.

### Surface Chirality Measured by Sum-Frequency Generation (SFG)

The main difference of SFG from SHG in the arrangement of setup is the use of a tunable IR laser that is organized into a vibrational spectroscopic tool, as the advantage over SHG-related approaches. In general, the measurement of C-SFG uses two polarized laser beams, one is visible and the other is tunable in IR, which are spatiotemporally overlapped at the surface/interface, and the SFG signal is coherently build up from the oriented molecules or chiral structures, which is then detected at the UV in s- or p-polarization by a charge-coupled device (CCD) or a photomultiplier (PMT). Since the chiroptical activities/effects are dispersive in nature, they depend on the probing wavelength, and thus, the spectrum of their measurements carries the signature of the configuration and conformation of the chiral structures. Similar to SHG-CD spectroscopy, the SFG spectroscopy, being one of the members of surface-specific nonlinear optical spectroscopic techniques, facilitates to record a spectrum for selected molecular bonding in a chiral substance and accurately characterizes the superficial molecular properties with polarized excitations. In this section, we will introduce the polarization-resolved vibrational SFG spectroscopy with literature studies from the following research groups, which have made substantial efforts to implement it on surface chirality.

**FIGURE 13:**
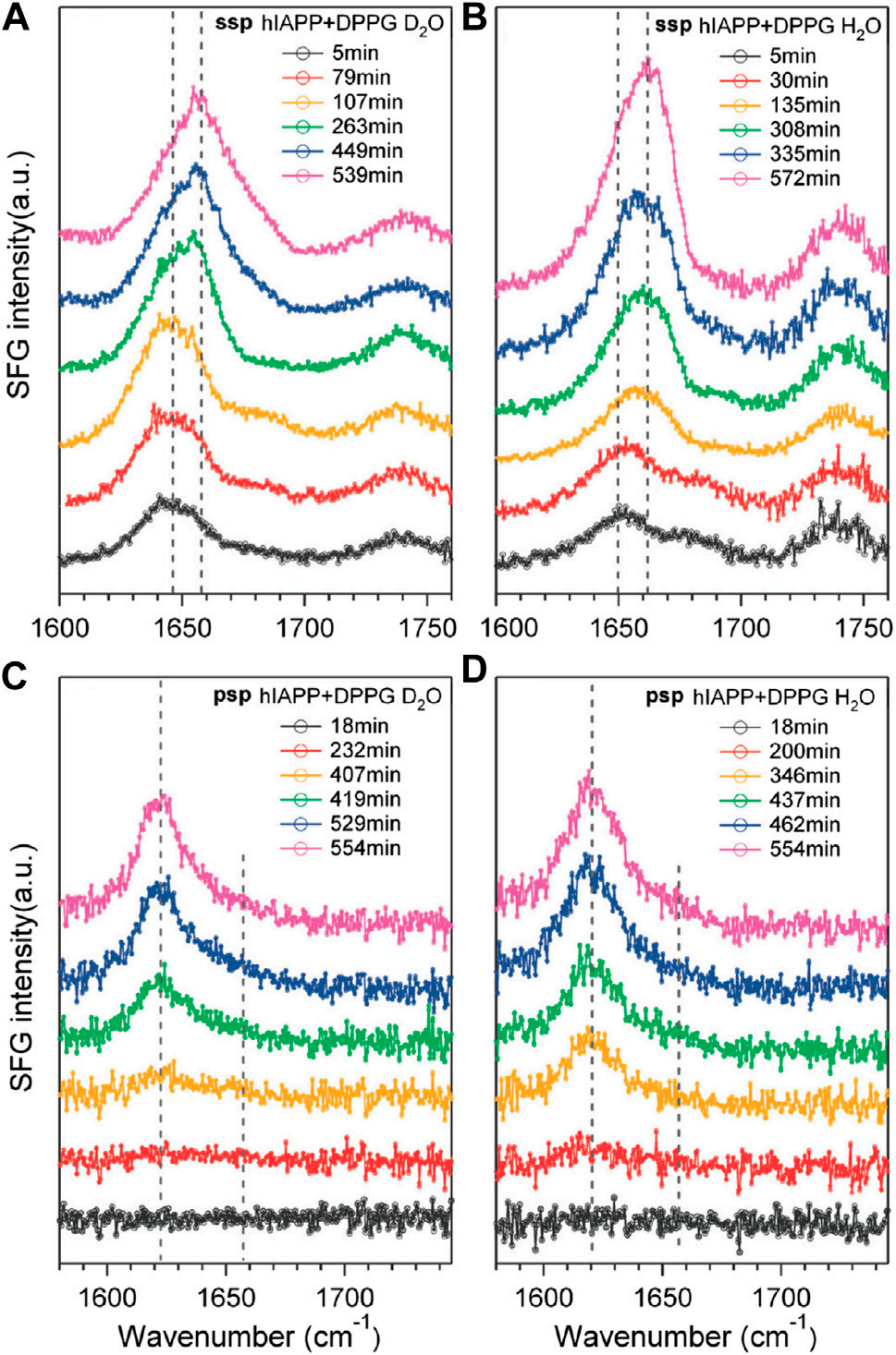
Time-dependent spectra of hIAPP taken using the ssp polarization in the presence of DPPG at the **(A)** air/D_2_O and **(B)** air/H_2_O interfaces. The time-dependent spectra of hIAPP taken using the psp polarization in the presence of DPPG at the **(C)** air/D_2_O and **(D)** air/H_2_O interfaces. The figure and caption have been adapted with permission from Fu et al., (2010), Copyright ^©^ 2010 American Chemical Society.

First, Yan’s research group has demonstrated various important experiments using the SFG vibrational spectroscopy in the last decade for the *in situ* and real-time characterization of protein secondary structures at interfaces (Fu et al., 2010; Fu et al., 2011a; Fu et al., 2014a). In these measurements, the secondary structures were investigated in the peptide N-H stretch and amide I bond of a series of model proteins, which were shown in terms of chiral and achiral SFG spectra. Related polarimetric measurement (psp polarization setting) can be used to classify various protein secondary structures and to obtain the orientation and folding kinetics of interfacial proteins. In the studies of amyloid aggregation at the lipid/water interface (Fu et al., 2010; Fu et al., 2011a), it is found that the amyloid protein has relatively disordered structures in normal states, while it aggregates into *β*-sheet structures (i.e., human islet amyloid polypeptide, hIAPP) on cell membranes in diseased states. The information helps to understand the early stages of amyloid aggregation on membrane surfaces. In experiments, combining C-SFG (psp polarization setting) and achiral SFG (ssp polarization setting) spectroscopic measurements in the amide I region at 1620 cm^−1^, the aggregation of hIAPP at the air/water interface was observed, as shown in Figure 13. Furthermore, the hIAPP solutions in a phosphate buffer with and without negatively charged phospholipid, dipalmitoylphosphoglycerol (DPPG), were used to confirm the induced amyloid aggregation. Result showed that DPPG molecules induce conformational changes in hIAPP instead of rIAPP found in the achiral spectra, while the lipid-induced parallel β-sheet structures can be found in the chiral spectra. It is demonstrated that only proteins folded into chiral secondary structures are able to be observed in C-SFG spectra without the interference of unfolded proteins and water.

On the other hand, the time-dependent chiral and achiral measurements of the interactions between hIAPP and DPPG at the air/water interface were also performed. In one of the experiments, the peptide N-H stretch vibrational band (∼3200 cm^−1^) was used to probe the aggregation kinetics (Fu et al., 2011a), in which the chiral specific signal was originated form an intermediate that formed and then disappeared before the parallel β-sheet structures being formed. Moreover, the time-dependent C-SFG signals in the N-H stretch and amide I vibrational regions showed opposite effects upon the addition of DPPG at the air/water interface. In another experiment (Fu et al., 2011a), the C-SFG spectra were obtained from three model systems, LKα14, pH-low insertion peptide (HLIP), and bovine rhodopsin, and they all showed the N-H stretch vibrational band. This N-H stretch signal is attributed to the *α*-helical structure constituted by hIAPP, as an intermediate in the initial aggregation of amyloid on membrane surfaces. Based on the above results, it is concluded that hIAPP shows a disordered structure when adsorbed onto the interface at the initial stage, and then, it is folded into *α*-helical structures before being converted into *β*-sheet structures of molecular aggregates.

For the separation of protein secondary structures at interfaces (Fu et al., 2011a; Fu et al., 2011b; Yan et al., 2014), a collection of C-SFG spectra of model peptides and proteins were taken for *α*-helix, 3_10_-helix, antiparallel *β*-sheet, parallel *β*-sheet (hIAPP), and disordered structures. Result showed that the C-SFG spectra of *α*-helix and 3_10_-helix exhibited the N-H stretch band, but no amide I band. However, both N-H stretch and amide I bands appeared in the C-SFG spectra of antiparallel *β*-sheets. On the contrary, the spectra of hIAPP aggregates only showed the amide I band. Based on the above results, C-SFG has unique selectivity to N-H stretch and amide I bands to distinguish parallel from antiparallel *β*-sheets and provides specific information for the vibrational structures of protein secondary structures. In addition, there is no amide I band signal from *α*-helixes in C-SFG spectra, and thereby, it is easier to deconvolute the amide I spectra. For other applications, the supramolecular structures, including orientation, symmetry, and chirality, can be extracted by the polarimetric measurements of C-SFG in combination with the analysis of hyperpolarizability tensor elements (Xiao et al., 2012). This kind of study allows for the derivation of amyloid orientation on membrane surfaces, which facilitates to understand the pathogenic mechanism of amyloid-related diseases at the early stages. Furthermore, it is able to shift eye on the C-H stretch vibrational mode (Wang et al., 2013), which is correlated with organic compounds. Therefore, C-SFG promises the observation of surface chirality not only on native membranes but also on synthetic biopolymers. Recently, from another research group, Kocsis et al., (2018) used this technique to discover the chirality induced by the dipolar orientation of confined water in chiral I-quartet artificial channels embedded in supported lipid bilayers similar to aquaporins shown in natural membrane channels. In experiments, achiral HC8 and chiral S-HC8 I-quartets were selected and embedded in supported lipid bilayers for C-SFG measurements on OH and N-H stretch bands. It was found in the chiral psp and spp spectra that a chiral ordering of confined water is formed in both HC8 and S-HC8 channels. Besides, the chirality of HC8 channel is lower than S-HC8 one mainly due to the enantiomeric distribution of the water wires that diminishes the C-SFG signal. In addition, Lu et al., (2019) used SFG vibrational spectroscopy to study the chirality from the peptide-Ca^2+^ ion interfacial interactions when fabricating 2D biomineral nanaosheets. Result showed that the C-SFG signal (psp polarization setting) of amide I band from *β*-sheet LE10 peptide centered at 1620 cm^−1^ was gradually increased with the interaction time and the CaC_2_O_4_ mineralization at the last step.

On the other hand, Wang’s research group has made a significant contribution to the application of spectrally and polarization-resolved C-SFG measurements. In their studies, a twin polarization angle approach was proposed to quantitatively analyze the surface chirality (Wei et al., 2009). In this approach, both the polarization directions of visible and IR laser beams are varied, and the C-SFG signal is detected at s- or p-polarization. The polarization-dependent SFG can be used to precisely measure the sign, relative phase, and absolute values of the chiral and achiral components in the *χ* ^(2)^ tensor and to identify the surface chirality with a DCE value. In experiments, the *S*- and *R*-limonene chiral liquid surfaces were measured, in which the DCE values obtained at 2878 cm^−1^ for *S*- and *R*-limonene liquid surfaces are 23.7 ± 0.4% and -25.4 ± 1.3%, respectively. It is noted that the detection at s-polarization is only sensitive to the small chiral contributions, similar to SHG-LD. Furthermore, since the C-SFG is sometimes lower than the experimental noise level or influenced by the interplay (leakage) of achiral SFG due to an imperfect polarimetric measurement, which cannot be precisely measured by traditional SFG vibrational spectroscopy, the same group used an interference (or heterodyne) method for the signal enhancement of surface chirality. It is realized by obtaining the interference spectra between the normal chiral and achiral spectrum when a ±*θ* degree of the p-polarization of the visible laser beam is introduced. Such kind of experiment is detailed in Wang et al. (2005), Fu et al. (2014b), Kocsis et al. (2018), Wang et al. (2019b).

**FIGURE 14:**
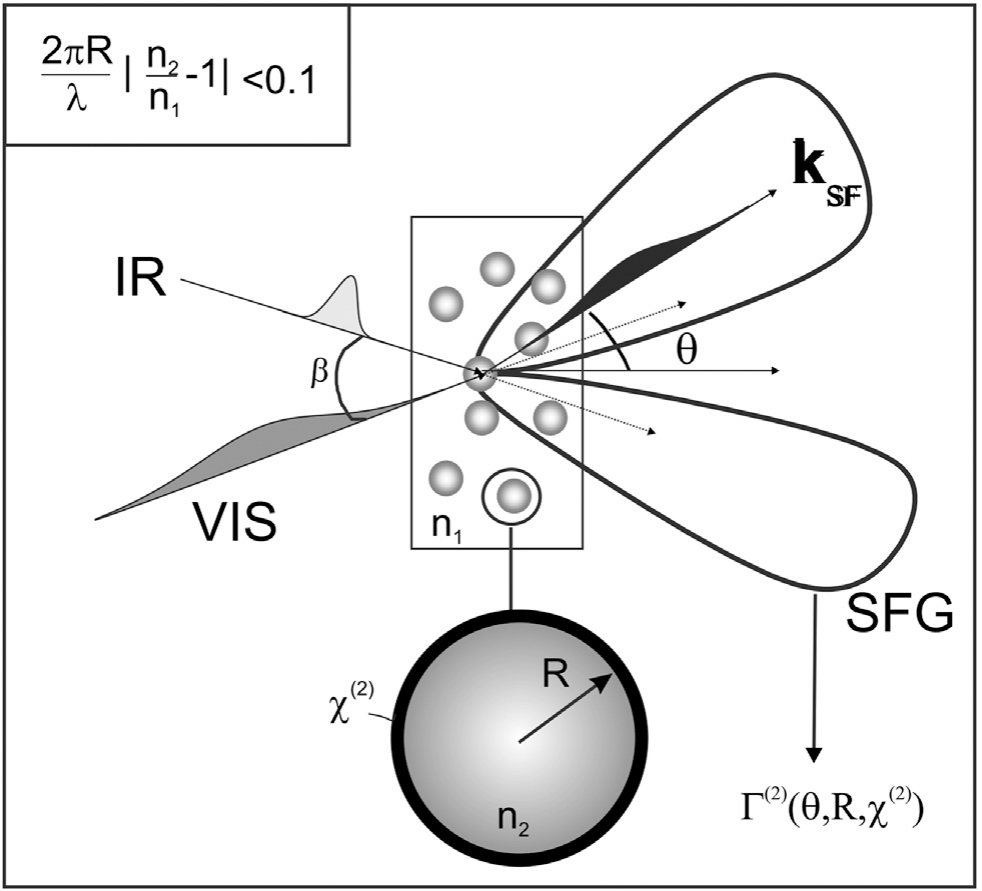
Illustration of a second-order vibrational sum-frequency scattering experiment. The incoming beams generate a nonlinear optical polarization at the interface of a particle. The polarization is the source of the scattered sum frequency light. The magnitude (represented by the scattering lobes) and spectral shape of the scattered field are determined by the molecular properties of the interface, which are contained in the second-order nonlinear susceptibility *χ* ^(2)^. The angular distribution reflects both the value of the second-order susceptibility and the radius of the particle and the scattering geometry. The criterium used in the Rayleigh–Gans–Debye formalism is depicted in the upper left corner. *n*_1_ and *n*_2_ are corresponding refractive indices. The figure and caption have been adapted with permission from de Beer and Roke, (2007), Copyright ^©^ 2007 The American Physical Society.

An important branch of SFG vibrational spectroscopy is used for the chiral detection of spherical surfaces, that is, NP surfaces (de Beer and Roke, 2007), which is pioneered by Roke et al. When the discussed geometry is transformed from planar to spherical surfaces, the procedure for deriving effective surface susceptibility should be different because the spatial definition of the incoming wave vectors with respect to the measurement geometry is changed. It indicates a difference in the transformation between the spherical (particle) coordinate of the local susceptibility and the Cartesian coordinate of the effective particle susceptibility. In general, the bulk C-SFG is easily detectable in the transmission mode due to the long coherent length of interaction that is dozens of times larger than that of the reflection mode or at a monolayer in transmission (Belkin et al., 2000), which needs to be enhanced with the help of chiral molecules adsorbed on the surfaces with a preferred orientation. Thus, the increased SFG signal is from certain *χ* ^(2)^ tensor elements of the adsorbates and the interactions with polarized excitations. Such kind of experiment uses linearly polarized laser beam, following specific polarization combinations as mentioned previously in the experimental scheme, as shown in Figure 14. For example, to measure surface chirality, the parameters of *β* (angle between the two beams) = 45°, *θ* (scattering angle) = 0^o^, and polarization combinations of pps, spp, and psp are used. In experiments, the isotropic spherical particles capped with a dielectric film were used, and the molecular interface properties were obtained by measuring the scattering patterns in conjugation with the SFG vibrational spectra. Importantly, the nonlinear Rayleigh–Gans–Debye scattering theory was developed (Roke et al., 2004; de Beer and Roke, 2007) to resolve the chirality and molecular orientation at NP surfaces. Furthermore, by considering the possible effect on the scattering pattern, the effective surface susceptibility as well as the SFG signal can be amplified by adjusting the experimental arrangement, for example, the angle between the two beams, *β*. Compared to the experiment of planar surfaces, the underlying second-order nonlinear scattering process is dissimilar with an SFG experiment in reflection mode. As a result, the superficial properties such as molecular orientation, composition, and chirality should be managed in a different way. Consequently, C-SFG can not only be used to measure the molecular properties of planar surfaces in reflection mode but also of NP surfaces in an aqueous suspension.

## Conclusion

In the quest for understanding chirality at different length scales, recent years have witnessed the development of a number of advanced chiroptical strategies, each having its unique advantages for enantiomeric recognition and quantification. In this mini review, we mainly focused on the analytical applications of contemporary chiroptical techniques that have been remarkably successful in interrogating chirality manifested at surfaces, membranes, and/or interfaces. Undoubtedly, with the capability of probing both molecular and surface chirality, linear chiroptical techniques, for example, ORD, ECD, VCD, and ROA, offer efficient platforms to study not only the identity and the absolute configuration of chiral molecular structures but also the dynamics of surface chirality including its genesis and amplification. That said, we note that a series of technical challenges must be addressed, as elaborated in a number of publications (Petrovic et al., 2015; Collins et al., 2017; Krupová et al., 2020), to further increase the detection sensitivity, acquisition speed, and spectral resolution. In many recent investigations, multimodal spectroscopic techniques have also been used to extract complementary details and/or cross validate the results (Buschhaus et al., 2010; Rivera-Fuentes et al., 2010; Duan et al., 2015b).

On the other hand, nonlinear chiroptical techniques have the potential to open new avenues to the understanding of chirality at surfaces by directly measuring OA effects from molecules localized at surfaces with excellent selectivity and specificity. The spectrally and/or polarization-resolved measurements of C-SHG and C-SFG have shown great potentials and analytical utility in studying surface chirality and dynamics of biological processes. Related applications have been found in chiral molecular recognition at surfaces/interfaces, characterization of protein secondary structures and orientation at interfaces, protein-membrane interactions, kinetics and thermodynamics of protein adsorption/binding at biological surfaces, and molecular aggregations at interfaces. In the near future, we expect that these nonlinear optical surface-specific tools can aid fundamental understanding of disease-induced chiral structure formation, effect evaluation of chiral drug at a particular step, and development of new surface molecular devices for epidemic prevention.
